# Inhibition Effects of Patchouli Alcohol, Carvacrol, *p*‐Cymene, Eucalyptol and Their Formulations Against Influenza Virus Pneumonia Through TLR4/NF‐κB/NLRP3 Signaling Pathway

**DOI:** 10.1111/cbdd.70150

**Published:** 2025-08-04

**Authors:** Ruilin Lv, Yi Li, Yinming Zhao, Qi Zhang, Xiaofang Wu, Xingyu Zhao, Linze Li, Ruying Tang, Jianjun Zhang, Linyuan Wang

**Affiliations:** ^1^ School of Chinese Materia Medica Beijing University of Chinese Medicine Beijing China; ^2^ Xinyang Vocational and Technical College Xinyang China; ^3^ Institute of Chinese Materia Medica China Academy of Chinese Medical Sciences Beijing China; ^4^ School of Traditional Chinese Medicine Beijing University of Chinese Medicine Beijing China

**Keywords:** antiviral activity, carvacrol, eucalyptol, inflammation, influenza virus type a, patchouli alcohol, *p*‐cymene, TLR4/NF‐κB/NLRP3 signaling pathway

## Abstract

As a kind of drug mostly used historically to treat epidemics, aromatic botanicals have volatile oils as active components. The study aims to evaluate the anti‐influenza viral pneumonia effects of volatile monomers patchouli alcohol (PA), carvacrol (CV), *p‐*Cymene (PC), eucalyptol (EC) and their formulations from various aspects through the influenza virus A/PR/8/34 (H1N1) infection experiment in vivo and in vitro and carry out in‐depth studies on the anti‐inflammatory mechanisms. In this study, we found that all four volatile monomers mentioned above could exert antiviral effects by suppressing pulmonary viral load and lung index and improving lung lesions in mice with influenza pneumonia. In addition, elevated levels of cytokines and chemokines in the serum were suppressed, the proportion of T‐lymphocytes in the peripheral blood was altered, and antioxidative stress indices were improved, whose mechanism of action related to anti‐inflammation, possibly acting on the Toll‐Like Receptor 4/Nuclear Factor‐κB/nucleotide‐binding domain leucine‐rich repeat and pyrin domain‐containing receptor 3 (TLR4/NF‐κB/NLRP3) pathway. The study provides an experimental basis for volatile monomers and their formulations of aromatic herbs for treating influenza virus pneumonia.

## Graphical abstract

The study provides an experimental basis for volatile monomers of aromatic herbs to be formulated for the treatment of influenza virus pneumonia. The overall scope of the study is shown in the image.

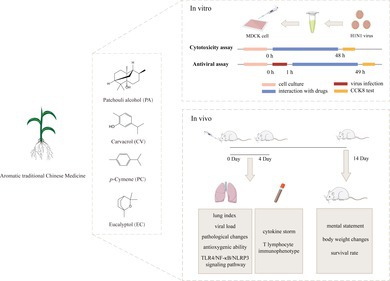

## 1 Introduction

Influenza is an acute respiratory infectious disease with a pathogen as influenza virus, of which influenza A virus is the most important (Hurt et al. 2004). Influenza virus pneumonia is an infectious disease caused by the influenza virus with lung inflammation as the main manifestation, which has a high mortality rate (Li, Wu, et al. 2018). The main clinical manifestations include fever and cough, and some patients are accompanied by gastrointestinal symptoms such as nausea and diarrhea, and critically ill patients may have symptoms like respiratory failure and death (Lee and Lee 2010). When influenza virus infects the respiratory tract, innate and acquired immune responses will be initiated to produce cytokines and chemokines (Droebner et al. 2008), with excess pro‐inflammatory cytokines and chemokines as well as innate immune cells reentering the lungs to create a “cytokine storm” which is the key for death from influenza virus pneumonia (Flagg et al. 2010). With 3–5 million severe cases of influenza worldwide each year, including 290,000–650,000 deaths (NIAC et al. 2023), influenza virus pneumonia remains an urgent problem worldwide.

Therapeutic strategies for this disease include synthetic chemical drugs that focus on symptomatic relief and antiviral therapy. However, the resistance to a single component has become increasingly prominent because of the high variability of influenza viruses (Elbahesh et al. 2019). Therefore, multicomponent herbal medicines are widely used in the long‐term treatment of the disease with the advantages of low adverse effects and low drug resistance (Wei et al. 2019; Yang et al. 2022), which mainly act through antiviral, anti‐inflammatory, and immune function regulation (Ma et al. 2018; Guo et al. 2021; Huang et al. 2021).

As an important part of the antiepidemic prescription of Chinese medicine, aromatic Chinese herbs are not only present in the classic antiepidemic prescriptions but also in the recommended prescriptions of the “Diagnosis and Treatment Protocol for Novel Coronavirus Pneumonia (Trial Version 9)” issued by the National Health Commission of the People's Republic of China. The compound formula “Dayuan Decoction” containing various aromatic Chinese herbs has a significant therapeutic effect on COVID‐19 (Zhang et al. 2020).

Studies on the Chinese herbal compound “Huo Xiang Su Ling Shuang Hua Yin” have shown that the product, especially the volatile oil component of the aromatic Chinese medicine in the product, has a significant effect against influenza A (H1N1) virus (Tang et al. 2022). Aromatic Chinese herbs have been used to prevent epidemics since ancient times, with their mechanism of action in the treatment of epidemics having been gradually unveiled in the modern era (Luo et al. 2020).

Volatile components are the main active ingredients of aromatic Chinese herbs. The obvious therapeutic advantages of aromatic Chinese herbs against influenza virus pneumonia have shed light on the study of volatile components of aromatic Chinese herbs. Some volatile components with research basis like patchouli alcohol (PA, from Pogostemonis Herba), carvacrol (CV, from Moslae Herba), *p*‐Cymene (PC, from Moslae Herba), and eucalyptol (EC, from Tsaoko Fructus) will be the study focus, all of which have promise to be drugs for the treatment of influenza virus pneumonia with antiviral and anti‐inflammatory properties (Li et al. 2012, 2016; Javed et al. 2021; Zhong et al. 2013). The method and criteria for screening the four monomers are described in Appendix A.

PA has an anti‐influenza virus effect, which protects host cells from viral infestation and attenuates systemic and pulmonary inflammatory responses (Li et al. 2012), possibly through modulation of the phosphatidylinositol 3 kinase/protein kinase B (PI3K/Akt) and extracellular regulated protein kinases/mitogen‐activated protein kinases (ERK/MAPK) signaling pathways (Yu et al. 2019). It can alleviate acute lung injury induced by lipopolysaccharide (LPS) with significant anti‐inflammatory effects (Yu et al. 2015).

CV has biological activities such as antiviral, antioxidative stress, and anti‐inflammatory (Javed et al. 2021). It can suppress IVA‐induced excessive immune response by inhibiting viral replication and Toll‐like receptor/retinoic acid‐inducible gene‐like receptor (TLR/RLR) pattern recognition (Zheng et al. 2021). CV exerts protective effects against benzo(a)pyrene‐induced lung injury by inhibiting lipid peroxidation, increasing antioxidant enzyme activities, and down‐regulating the expression of inducible nitric oxide synthase (iNOS), nuclear factor‐κB (NF‐κB) and Cyclooxygenase‐2 (COX‐2) inflammatory marker proteins in lung tissues (Barnwal et al. 2018).

PC has pharmacological activities such as antioxidant, anti‐inflammatory, antiparasitic, antiviral, and antibacterial (Balahbib et al. 2021). It may affect herpes simplex virus‐1 (HSV‐1) replication by interacting with the viral envelope before viral adsorption (Sharifi‐Rad et al. 2017). The anti‐inflammatory effect of PC was significant against carrageenan‐induced pleurisy, which inhibited the recruitment of neutrophilic granulocyte (NE) and total leukocyte count (TLC) into the pleural cavity and reduced the level of Tumor Necrosis Factor‐α (TNF‐α) in pleural exudate and NO production by macrophages (de Santana et al. 2015). It also exerts a protective effect against LPS‐induced acute lung injury (Xie et al. 2012), which may be related to the anti‐inflammatory effect by blocking the NF‐κB and MAPK signaling pathways (Zhong et al. 2013).

EC, also known as 1,8‐Cineole, mainly has anti‐inflammatory, antibacterial, antiviral, and antioxidant activities, which have therapeutic effects on a variety of respiratory diseases, such as influenza, bronchitis, pneumonia, asthma, and chronic obstructive pulmonary disease (COPD), and has entered clinical trials in some cases (Cai et al. 2021). EC has a protective effect against influenza virus A/FM/47 (H1N1) infection in mice. It can effectively reduce the levels of cytokines and chemokines in nasal lavage and lung tissue and the expression of NF‐κB p65, ICAM‐1, and VCAM‐1 in lung tissues and enhance the protection against influenza virus (IFV)‐infected mice by attenuating the inflammatory response of the lungs (Li et al. 2016). Results of a clinical trial suggest that eucalyptus oil extract relieves asthma and COPD through its anti‐inflammatory effects (Juergens et al. 2003).

However, single‐target drugs have had limited success in treating multifactorial diseases, and the development of multicomponent Chinese medicine is becoming the forefront of new drug development, where ideas and methods are being innovated (Zhang et al. 2015; Liang et al. 2022). The study attempts to apply the formulations of the above four monomer components according to the multicomponent Chinese medicine development methodology to develop a novel formulation against influenza virus pneumonia, verify the potentiation and toxicity reduction effects of formulations through in vivo and in vitro experiments, and investigate in depth the related anti‐inflammatory pathways that it might regulate.

The innovation of this study lies in the first combination of these four monomers to investigate their inhibitory effects on influenza virus pneumonia, and the in‐depth exploration of the mechanisms of action of these substances. Previous studies may not have focused on the role of these substances in this signaling pathway. This study provides a new molecular mechanism basis for understanding their pharmacological effects. Unlike typical studies on single components, this study also considers the potential synergistic or antagonistic effects between multiple components, which is more in line with the practical application of drugs with multiple components exerting their effects. This provides a theoretical basis for the development of multitarget, multicomponent anti‐influenza virus pneumonia drugs or formulations. These individual components are mostly derived from natural plants. Studying their inhibitory effects on influenza virus pneumonia can help promote the application of natural products in the field of antiviral drug development, provide a scientific basis for the development of safe and effective anti‐influenza virus pneumonia drugs from natural resources, and also provide new avenues for the deep development and utilization of natural products.

## 2 Materials and Methods

### 2.1 Chemical and Reagents

Patchouli alcohol (purity 99.63%, RFS‐B03702012009, Chengdu Herbpurify Co. Ltd.; Chengdu, China); carvacrol (purity 99.35%, RFS‐X04811812016, Chengdu Herbpurify Co. Ltd.; Chengdu, China); *p*‐Cymene (purity 98.94%, D03711809025, Chengdu Herbpurify Co. Ltd.; Chengdu, China); eucalyptol (purity 99.48%, RFS‐A00311812016, Chengdu Herbpurify Co. Ltd.; Chengdu, China); oseltamivir phosphate capsules (0222209003, Yichang HEC Changjiang Pharmaceutical Co. Ltd.; Yichang, China); Dimethyl sulfoxide (DMSO) (r21075231, Bioruler; Nantong, China); Tween 80 (P6474, Sigma‐Aldrich; the USA).

### 2.2 Cell Culture and Virus Preparation

The Madin‐Darby canine kidney (MDCK) cell line was obtained from the Beijing Institute of Microbiology and Epidemiology. MDCK cells were cultured in a medium (MEM) of 10% fetal bovine serum and 1% penicillin–streptomycin in an incubator at 37°C with 5% CO_2_. MDCK cells with good growth status were diluted into a single‐cell suspension with cell culture medium, inoculated into a 96‐well cell culture plate at a density of 2 × 10^4^ cells/well, and cultured in a culture incubator at 37°C with 5% CO_2_ for 20 h–24 h until the cells grew into monolayers.

The Beijing Institute of Microbiology and Epidemiology provided human influenza virus strain A/PR/8/34 (H1N1), which was propagated in chicken embryos and stored at −80°C for further use.

### 2.3 Virus Infection In Vitro

For in vitro infections, influenza virus solution was diluted to 100‐fold 50% Tissue culture infective dose (TCID50) with virus culture solution (virus culture solution: MEM + 1% bicinchoninic acid method (BSA) + 1% Penicillin–Streptomycin (P/S) + 2 μg/mL TPCK‐trypsin) and infected with MDCK cells that had grown into monolayers for 1 h.

### 2.4 Therapeutic Index (TI) Determination

The experimental drugs were prepared into different mother liquors with Dimethyl sulfoxide (DMSO) before the experiment. Steps for drug solution preparation are shown in Appendix B. During the experiment, the cell culture medium was removed and the drug mother liquor was diluted to 200 μM with MEM medium, and continued to be diluted according to the 2‐fold ratio, followed by adding into the 96‐well plate with 8 concentration gradients for culturing 48 h. After that, the drug‐containing medium was aspirated, and the Cell Counting Kit‐8 (CCK8) (P2011433, Adamas life; Shanghai, China) was added to detect cell survival, with the cell survival rate and 50% toxic concentration (TC50) calculated by the formula of Cell survival rate (%) = (optical density (OD) value of drug group‐OD value of control group) / (OD value of normal cell group‐OD value of control group) × 100%.

The virus‐containing medium was aspirated after 1 h of cell infection, and drug‐containing medium was added in the same manner as described above for culturing 48 h, followed by applying CCK8 reagent to measure absorbance values of each well, and calculating the virus inhibition rate, the 50% inhibitory concentration (IC50), and the therapeutic index (TI) by the formula of Virus inhibition rate (%) = (OD value of drug group‐OD value of virus model group)/(OD value of normal cell group‐OD value of virus model group), TI = TC50/IC50.

### 2.5 Virus Infection In Vivo

#### 2.5.1 Mortality Protection Experiment

For in vivo infection, male BALB/c mice (6–8 weeks old with 16–18 g purchased from SPF (Beijing) Biotechnology Co. Ltd., Certificate No. SCXK (Beijing) 2019–0010) were randomly grouped with 10 in each group after 7 days of adaptive feeding, with the grouping and the administration dosage shown in Table 1. To demonstrate the efficacy of the drug, the experiment requires modeling. In this experiment, modeling is done by nasal drops of diluted influenza virus. In order to make the degree of infection in mice in a suitable range, it is necessary to find the LD50 of the infected virus mice first, and generally use 2–10 times the LD50 of the influenza virus for modeling (Guo et al. 2021; Huang et al. 2024; Li et al. 2024). BALB/c mice were intranasally infected with H1N1 (5LD50/each) for survival experiments. After 2 h of infection, the drug was administered each time by gavage at 0.1 mL/10 g once daily for 4 d, with the normal and model groups gavaged with distilled water under the same conditions for 14 d. The day of virus inoculation was defined as Day 0. Daily gavage, changes in body weight, mental status, and death of mice were recorded. Drug efficacy experiment: Several identical male BALB/c mice were randomly grouped with 10 in each group, and the drug was administered in the same way as described above after 2 h of intranasal infection. On the 4th day after the infection, the mice's eyeballs were removed to collect blood, followed by executing mice to take lungs. The lung index was calculated by the formula of lung index = lung tissue mass (g)/body mass (g) × 100%. All animal experiments were approved by the Animal Care Committee of Beijing University of Chinese Medicine (Ethics No. BUCM‐4‐2,022,040,101‐2001, BUCM‐4‐2,022,062,801‐2094), and all viral experiments were carried out in an animal biosafety shelter laboratory‐2 (ABSL‐2) biosafety chamber.

**TABLE 1:** Animal grouping and oral dosage.

Group	Composition and dosage
PA	PA 20 mg/kg/d
CV	CV 25 mg/kg/d
PC	PC 8.33 mg/kg/d
EC	EC 100 mg/kg/d
PEC‐L	PA 5 mg/kg/d + EC 25 mg/kg/d + CV 6.25 mg/kg/d
PEC‐M	PA 10 mg/kg/d + EC 50 mg/kg/d + CV 12.5 mg/kg/d
PEC‐H	PA 20 mg/kg/d + EC 100 mg/kg/d + CV 25 mg/kg/d
PECP‐L	PA 5 mg/kg/d + EC 25 mg/kg/d + CV 6.25 mg/kg/d + PC 2.08 mg/kg/d
PECP‐M	PA 10 mg/kg/d + EC 50 mg/kg/d + CV 12.5 mg/kg/d + PC 4.16 mg/kg/d
PECP‐H	PA 20 mg/kg/d + EC 100 mg/kg/d + CV 25 mg/kg/d + PC 8.33 mg/kg/d

### 2.6 Histological Analysis

After the mice were executed, lung tissues were collected to fix in 4% paraformaldehyde, followed by being dehydrated and embedded in paraffin for preparing tissue sections, which were finally stained with hematoxylin and eosin (H&E). The pathological changes of lung tissue were observed under an optical microscope (a magnification of 100 times).

### 2.7 qRT‐PCR Analysis

Total RNA was extracted from mouse lung tissues stored at −80°C by centrifugation in a tissue grinder. mRNA of the H1N1 virus nucleoprotein (NP) gene was reverse transcribed using SweScript All‐in‐One RT SuperMix for qPCR (One‐Step gDNA Remover) kit. The relative expression of the H1N1 NP gene was detected by adding a synergetic binding reagent (SYBR) Green quantitative polymerase chain reaction (qPCR) Master Mix. The relative expression of NP gene mRNA was analyzed by the 2^−ΔΔCt^ method using glyceraldehyde‐3‐phosphate dehydrogenase (GAPDH) as an internal reference. The primers were designed and synthesized by Wuhan Servicebio Technology Co Ltd., with the primer sequences shown in Table 2.

**TABLE 2:** Primer sequence for RT‐PCR.

Target gene	Sequence (5′‐3′)
IAV‐NP‐F^a^	GTCAGAATGATCAAACGTGGGA
IAV‐NP‐R^b^	TACGGCAGGTCCATACACACAG
GAPDH‐F^c^	CCTCGTCCCGTAGACAAAATG
GAPDH‐R^d^	TGAGGTCAATGAAGGGGTCGT

^a^ IAV‐NP‐F: Forward primer of nucleoprotein gene in influenza virus A.

^b^ IAV‐NP‐R: Reverse primer of nucleoprotein gene in influenza virus A.

^c^ GAPDH‐F: Forward primer of Glyceraldehyde‐3‐Phosphate Dehydrogenase gene.

^d^ GAPDH‐R: Reverse primer of Glyceraldehyde‐3‐Phosphate Dehydrogenase gene.

### 2.8 Enzyme‐Linked Immunosorbent Assay

Serum samples were separated by centrifugation at 3500 r/min (r: revolutions) for 15 min at 4°C. Then, the serum levels of TNF‐α, Interferon‐γ (IFN‐γ), Interleukin‐2 (IL‐2), Interleukin‐6 (IL‐6), Interferon‐inducible Protein‐10 (IP‐10), Monocyte Chemoattractant Protein‐1 (MCP‐1), and Macrophage Inflammatory Protein‐1α (MIP‐1α) were measured using commercially available enzyme‐linked immunosorbent assay (ELISA) kits according to the manufacturer's instructions, which were purchased from Shanghai Enzyme‐linked Biotechnology Co. Ltd.

### 2.9 Flow Cytometry Analysis

The ratio of human cluster of differentiation 4 (CD4^+^) T cells and human cluster of differentiation 8 (CD8^+^) T cells subgroups was determined by flow cytometry, which means 100 μL of peripheral blood was anticoagulated with heparin and added with fluorescein isothiocyanate (FITC)‐labeled CD8^+^ antibody and phycoerythrin (PE)‐labeled human cluster of differentiation 3 (CD3^+^) antibody (1:100 dilution) to incubate for 25 min at room temperature and protected from light, followed by washing with PBS for 3 times and centrifuging to discard the supernatant. Then, the cells were resuspended by 100 μL of phosphate‐buffered saline (PBS) and analyzed by flow cytometry (ACEA NovoCyte, ACEA Biological (Hangzhou) Co. Ltd.; Hangzhou, China), followed by carrying out lymphocyte subpopulation analysis using NovoExpress 1.5.0 flow cytometric analysis software, with the percentage of CD4^+^T cells subgroup expressed by the ratio of CD3^+^CD4^+^T cells subgroup, and the percentage of CD8^+^T cells subgroup by the ratio of CD3^+^CD8^+^T cells subgroup, with CD4^+^T/CD8^+^T values calculated. FITC‐labeled CD4 and CD8 antibodies and PE‐labeled CD3 antibody were purchased from BD, USA.

### 2.10 Determination of Biochemical Indices Like SOD, MDA, G,SH‐PX and ROS

The levels of total superoxide dismutase (T‐SOD), malondialdehyde (MDA), glutathione peroxidase (GSH‐Px), and reactive oxygen species (ROS) in the lung tissue homogenate of each group of mice were determined by ELISA, with the specific operation carried out according to the instructions of the corresponding kits. The T‐SOD, MDA, and GSH‐Px kits were purchased from Nanjing Jiancheng Bioengineering Research Institute. The ROS kit was purchased from Beijing Kangjia Hongyuan Biotechnology Co. Ltd.

### 2.11 Western Blot Analysis

Proteins from lung tissues were lysed in radioimmunoprecipitation assay (RIPA) lysate to obtain protein lysates, which were centrifuged to remove insoluble compounds, followed by applying bicinchoninic acid method (BCA) Protein Quantification Kit (G2026, Applygen; Shanghai, China) to quantify samples. Protein samples were separated on a 10% SDS‐PAGE gel, and the protein blot was transferred onto a 0.45 μm PVDF membrane (IPVH00010, Millipore; USA). The polyvinylidene fluoride (PVDF) membrane was closed with 5% BSA‐TBS with Tween‐20 (TBST) for 1 h at room temperature and incubated with primary antibody (dilution ratio 1:1000) overnight at 4°C. The mentioned primary antibodies included GAPDH (5174, CST; USA), nucleotide‐binding domain leucine‐rich repeat and pyrin domain‐containing receptor (NLRP3) (315,101, CST; USA), TLR4 (19811‐1‐AP, Proteintech; USA), NF‐κB p65 (8242, CST; USA), phospho‐NF‐κB p65 (p‐NF‐κB p65) (3033, CST; USA), Caspase1 (124,232, CST; USA), myeloid differentiation primary response protein 88 (MyD88) (4283, CST; USA), and apoptosis‐associated speck‐like protein containing a caspase‐recruitment domain (ASC) (67,824, CST; USA). Protein blots were visualized using 5% BSA‐TBST diluted with the secondary antibody of goat anti‐rabbit IgG (H + L) HRP (111–035‐003, Jackson; USA) (dilution ratio 1:10,000), incubated at room temperature for 1 h, and membranes were exposed to enhanced chemiluminescence (ECL) reagent (abs920, Absin; Shanghai, China). Relative gray values of different proteins were analyzed using the software Image‐PRO IPP6.

### 2.12 Statistical Analysis

The results were presented as mean ± standard deviation (SD) and compared by a two‐tailed unpaired Student's t test or a one‐way ANOVA test. All data were visualized using GraphPad Prism, and *p* < 0.05 was considered statistically significant.

All experimental data were expressed as the means ± SD. Statistical analysis was carried out using the SPSS 20.0 software in a one‐way analysis of variance (ANOVA) followed by the Least‐Significant Difference (LSD) post hoc test or the Dunnett T3 test for comparison of multiple groups. *p* < 0.05 was considered statistically significant.

## 3 Results

### 3.1 Antiviral Studies of Four Volatile Monomers In Vitro

**FIGURE 1:**
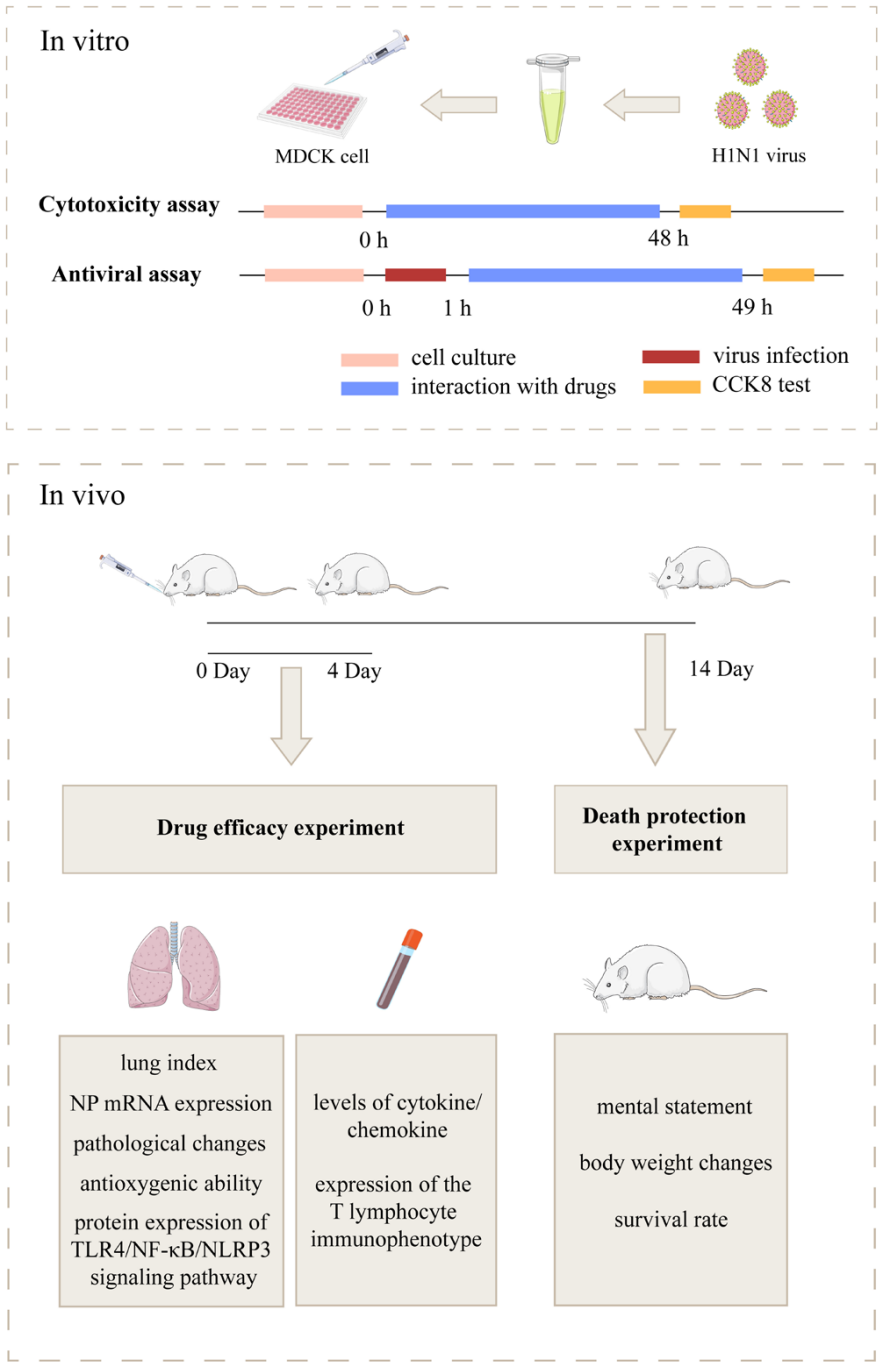
The scheme of exploring the anti‐influenza virus effect mechanisms of patchouli alcohol (PA), carvacrol (CV), *p‐*Cymene (PC), eucalyptol (EC) and their formulations. The experiments in vitro demonstrated the administration time and operation steps of cytotoxicity and antiviral assays, while the experiments in vivo showed the administration days and sampling nodes of mice, as well as the categories of detection indicators.

**FIGURE 2:**
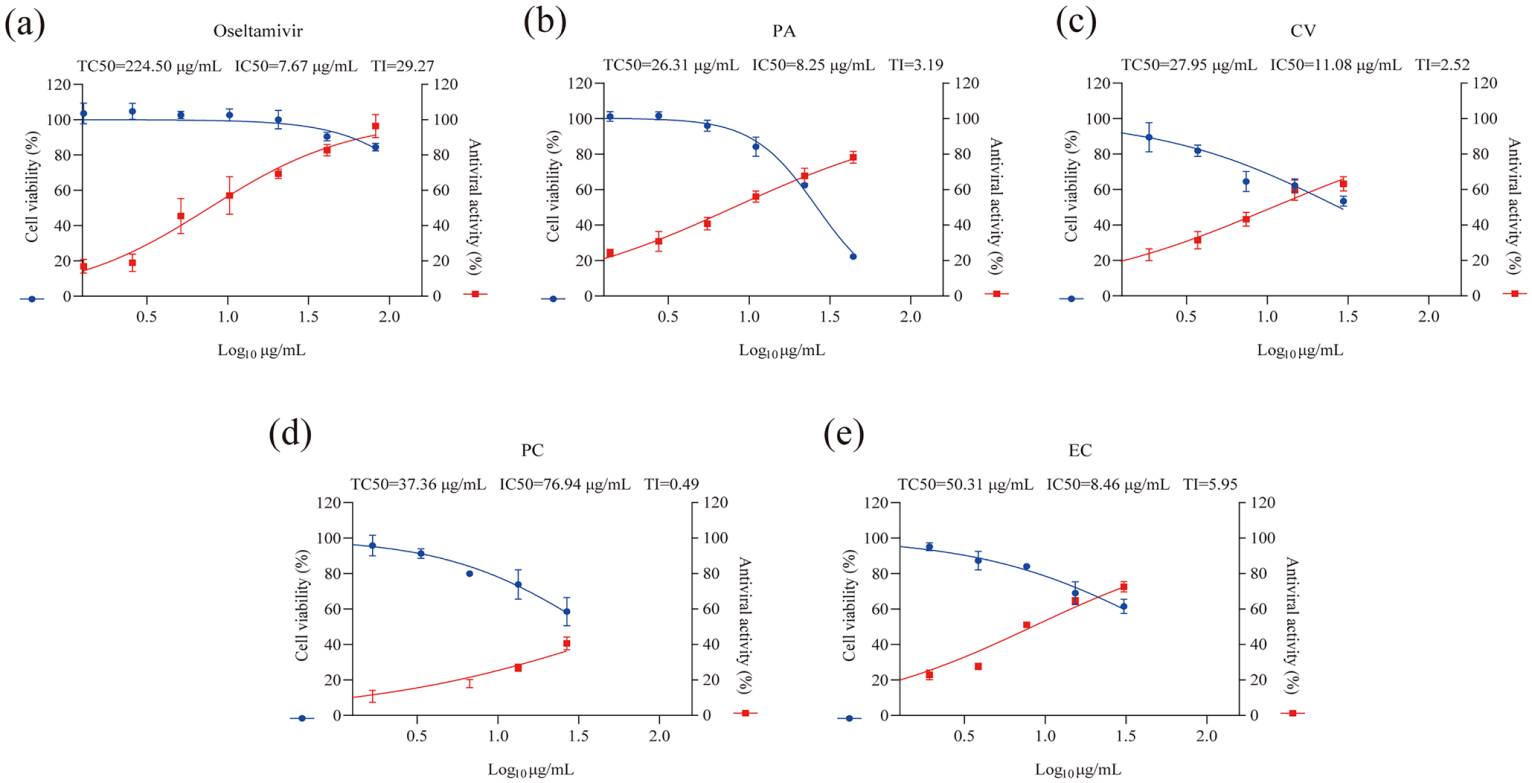
In vitro assays. Cytotoxicity and antiviral activity of (a) Oseltamivir (positive control drug), (b) PA, (c) CV, (d) PC and (e) EC in Madin‐Darby canine kidney (MDCK) cells (*n* = 3).

The study was divided into two parts: In vitro and in vivo experiments. Figure 1 shows study procedures. In vitro experiments can preliminarily explore the antiviral effect of monomers. In order to observe the inhibitory effects of the four monomers on the influenza virus in MDCK cells, the cell viability was detected in the experiment using the CCK‐8 reagent, which was used to indirectly reflect the drug toxicity and the inhibitory effects on the virus. The purpose of calculating TI is to indicate the risk taken by the drug in exerting its therapeutic effect, and it is generally considered that a drug with a larger TI is more practical, which was in accordance with the purpose of the experiment to develop a safe and effective multicomponent Chinese medicine. The four monomers showed different degrees of cytotoxic effects and inhibitory effects on viruses, respectively (Figure 2), with the therapeutic indices of the anti‐influenza viruses of PA, CV, PC, and EC being 3.19, 2.52, 0.49, and 5.95, respectively. Comparatively speaking, PC has a weaker inhibitory effect on viruses with higher toxicity, while EC has characteristics of low toxicity and high efficacy.

### 3.2 Effects of PA, CV, PC, and EC on the Survival of H1N1‐Infected Mice

**FIGURE 3:**
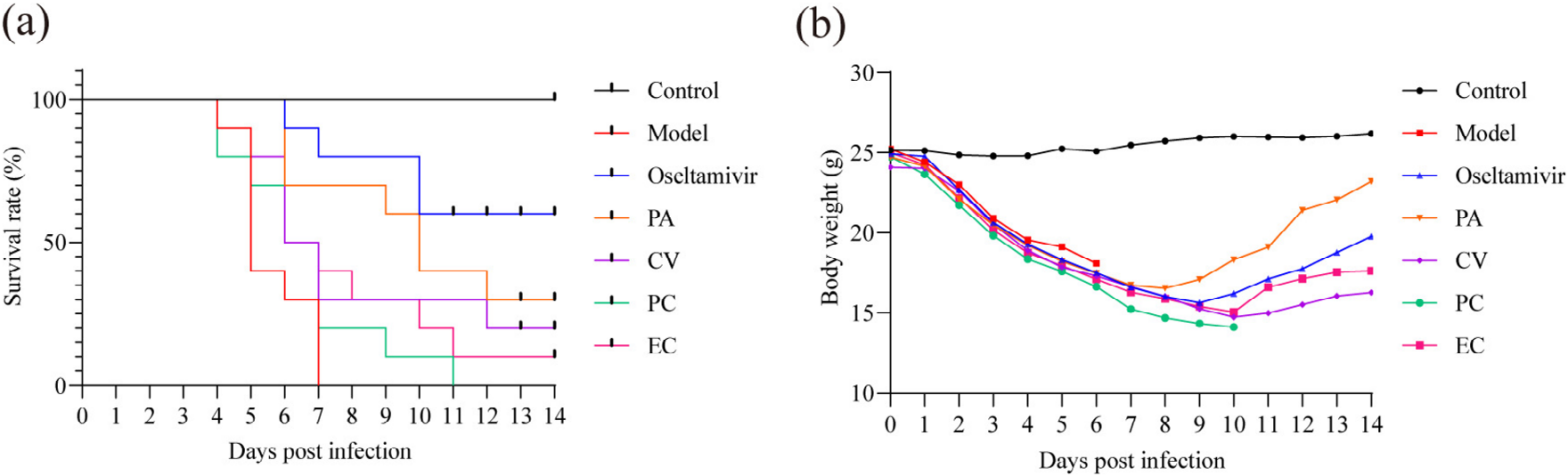
Survival test results of monomers. (a) Effect of different groups on the survival rate of H1N1‐infected mice (*n* = 10). (b) Average body weight changes of mice among different groups during the survival test.

In order to determine the protective effects of four monomers against H1N1 infection in vivo, BALB/c mice 6–8 weeks old were intranasally infected with 5LD50 H1N1 influenza virus, with the death protective effect of the drugs reflected by observing the survival rate and survival time of the mice. The control drug oseltamivir is a neuraminidase inhibitor (Javanian et al. 2021), which is often used as a positive control in anti‐influenza virus drug studies due to its high efficiency and low toxicity (Cheng et al. 2023; Huang et al. 2024; Li et al. 2024). Therefore, oseltamivir was chosen to use as a positive control in this experiment. After infection, the mice continued to lose weight and showed obvious emaciation, assemble, humpback, shudder, cough, squinting, reversed hair, and slow movement. Some mice began to die on the 4th day after infection, and all mice in the model group died on the 7th day after infection (Figure 3). The overall survival time of the administered groups was prolonged, and the surviving mice started to regain their body weights 7–10 days after infection, with the survival rates of the oseltamivir, PA, CV, PC, and EC groups being 60%, 30%, 20%, 0%, and 10%, respectively, after infection with a lethal dose of the H1N1 virus for 14 days. This study found that all four monomers had inhibitory effects on H1N1 virus and improved mouse survival rates to varying degrees.

### 3.3 Protective Effects of PA, CV, PC and EC Against Acute Lung Injury Caused by H1N1 Infection

**FIGURE 4:**
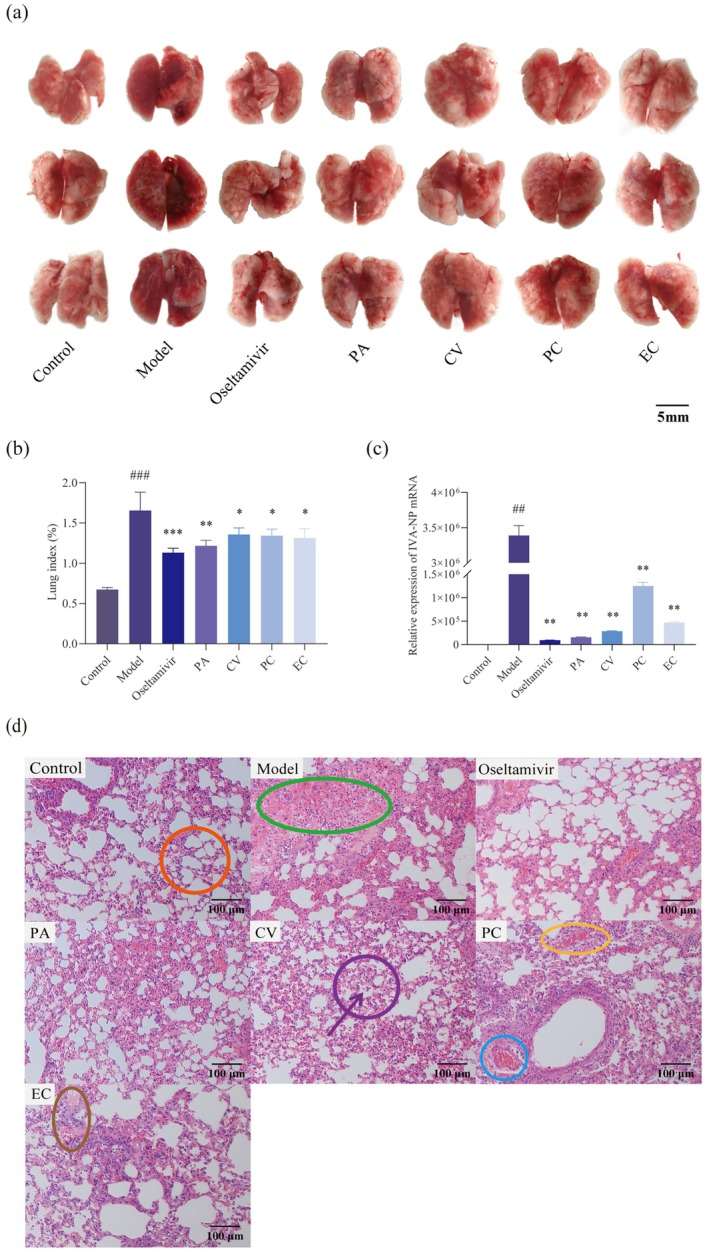
Effects of PA, CV, PC and EC on lung in H1N1‐infected mice. (a) Gross macroscopic examination of lungs in different groups. Changes of (b) lung index (*n* = 10) and (c) relative expression of Influenza Virus A nucleoprotein (IVA‐NP) mRNA (*n* = 3) in lung tissue among groups. (d) Pathological changes of lung tissues following H&E staining. The bright yellow circle shows pulmonary hemorrhage. The blue circle shows the dilated and congested blood vessels. The dark yellow circle shows a pink transparent film formed by serous exudation. The orange circle shows normal alveoli. The purple circle shows collapsing alveoli. The purple arrow points to the exudation of inflammatory cells. The green circle shows collapsed alveoli. Data are presented as the means ± SD. *p < 0.05, **p < 0.01, ***p < 0.001 versus the model group. #*p* < 0.05, ##*p* < 0.01, ###*p* < 0.001 versus the control group.

The lung is a key target organ for H1N1 influenza virus infection. The study confirmed the therapeutic effects of four monomers on mice with influenza virus pneumonia by observing the lung index, pulmonary virus load, gross lesions of lung tissues, and histopathological changes in the lungs. The lung index can directly reflect the degree of pulmonary edema in mice, which is caused by the excessive increase of tissue fluid in the lungs or even infiltration into the pulmonary alveolar. Pulmonary edema is a state of macroscopic lung tissue that becomes enlarged by edema, which is assessed by the amount of lung index variation. Inflammatory exudation in the alveoli increases the mass of the lung, so the lung index can be used as a common evaluation index of lung lesions and treatment effects, which can objectively reflect the degree of change of inflammatory lesions in the lungs (Guo et al. 2021; Wang et al. 2023; Cheng et al. 2023). The results showed that the lung index of mice in the administered group was significantly lower (*p* < 0.01 or *p* < 0.05) than the model group (Figure 4a). The core of the influenza virus is the viral ribonucleoprotein complexes (vRNPs), which are the smallest functional units of the viral genome's transcription and replication. Nucleoprotein (NP) is one of the important components of vRNPs and plays a key role in the entire life cycle of the influenza virus. There are various host factors in the organism that affect virus proliferation through NP (Eisfeld et al. 2015; Wang et al. 2017; Watanabe et al. 2014). Therefore, the relative expression of the NP gene can be used as a marker not only to indirectly reflect the amount of influenza virus in the lung tissue of mice but also how much replication potential the virus has. The relative expression of viral NP gene mRNA in mouse lung tissues was determined by Real‐Time Quantitative Reverse Transcription Polymerase Chain Reaction (qRT‐PCR) to observe the drug effect in inhibiting virus replication in mice. The viral load here refers to the relative expression level of the NP gene. Reduction of pulmonary viral load in mice implies the inhibitory effect of the drug on the influenza virus in mice. The pulmonary virus load of the model group was significantly higher than the control group (*p* < 0.01). The elevation of pulmonary virus load was inhibited in all monomers compared to the model group (*p* < 0.01), with the inhibitory effect being most pronounced for PA and relatively weak for PC (Figure 4b), which was in accordance with the performance in the cellular experiments described above. The lung tissues of the model group were markedly edematous, dark red, and extensively hemorrhagic. The comparison results showed that the morphology of lung tissues improved to different degrees after the administration, with lighter color of lungs and reduced areas of pulmonary edema and pulmonary hemorrhage indicating the alleviation of lung lesions (Figure 4c). Bright yellow circles indicated pulmonary hemorrhage. Under the light microscope, the histopathological changes in the lung tissue of mice showed that the alveolar structure of the normal group was the same, and the morphology of the lung tissue was normal. The alveolar interstitium includes connective tissue, blood vessels, lymph nodes, and nerves. In the model group, the alveolar interstitium was significantly widened, with dilated and congested blood vessels (in the blue circle), fractured pulmonary alveolar walls, loss of alveolar structure, a large number of inflammatory cells infiltrating and exuding, and large lung consolidation in almost the whole lung. Lung parenchyma is all levels of bronchial branches and alveoli within the lungs. Pulmonary consolidation in this study is a pathologic change that results in the loss of gas in the lungs due to a large amount of inflammatory exudate filling the alveolar cavities. Pneumonia manifested as inflammatory exudation, with exudation of tissue fluid forming a hyaline membrane (in the dark yellow circle), and exudation of inflammatory cells filling the spaces in the alveoli that were ventilated (in the direction of the purple arrow), leading to the collapse of the alveoli. Normal alveoli were shown in the orange circle, purple circles indicated alveoli that were collapsing, and a portion of lung consolidation showed alveoli that had already collapsed, as shown in the green circle in the figure. In the administration group, the degree of lesions was significantly reduced, as evidenced by the limited extent of lesions, lower density of inflammatory cells, less plasma exudation in the alveolar cavity, and a more intact alveolar structure (Figure 4d). The above results showed that the four monomers had therapeutic effects on influenza virus pneumonia and PA has better results.

### 3.4 Inhibitory Effects of PA, CV, PC, and EC on Cytokine Storm

**FIGURE 5:**
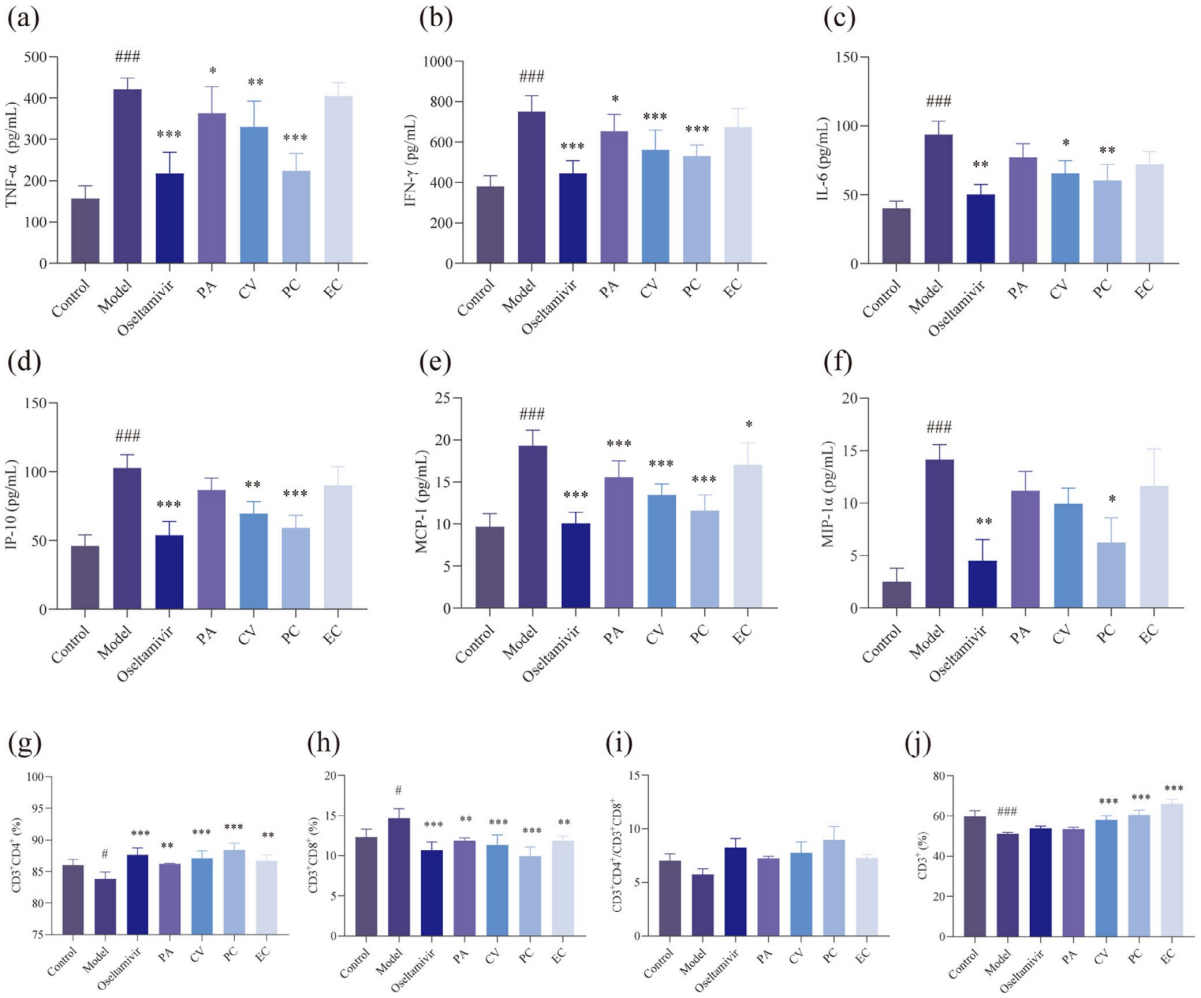
Effects of PA, CV, PC, and EC on levels of cytokine/chemokine and the expression of the T lymphocyte immunophenotype in blood in H1N1‐infected mice. Levels of (a) Tumor Necrosis Factor‐α (TNF‐α), (b) Interferon‐γ (IFN‐γ), (c) Interleukin‐6 (IL‐6), (d) Interferon‐inducible Protein‐10 (IP‐10), (e) Monocyte Chemoattractant Protein‐1 (MCP‐1), (f) Macrophage Inflammatory Protein‐1α (MIP‐1α) in serum were determined using ELISA kits (*n* = 7). Changs in (g) CD3^+^CD4^+^/CD3^+^CD8^+^, the percentage of (h) CD3^+^CD4^+^, (i) CD3^+^CD8^+^ and (j) CD3^+^ in blood between groups detected by flow cytometry assay (*n* = 3). Data are presented as the means ± SD. *p < 0.05, **p < 0.01, ***p < 0.001 versus the model group. #*p* < 0.05, ##*p* < 0.01, ###*p* < 0.001 versus the control group.

Mouse serum was collected at the end of the experiment to detect cytokine changes by ELISA, including levels of cytokines TNF‐α, IFN‐γ, IL‐6, and chemokines IP‐10, MCP‐1, MIP‐1α. As shown in the Figure 5a–f, the four monomers and oseltamivir were able to effectively inhibit the production of cytokine storm due to the invasion of influenza virus, as evidenced by the effective inhibition of cytokine TNF‐α, IL‐6, and INF‐γ, while at the same time, the chemokines IP‐10, MCP‐1, and MCP‐1α were all correspondingly decreased. Among the four monomers, PC had significant regulatory effects on cytokines and prominent anti‐inflammatory effects.

### 3.5 Immunoregulation of T Lymphocytes in H1N1‐Infected Mice by PA, CV, PC, and EC

To investigate the effects of four monomers on cellular immunity in infected mice, the proportions of the T‐lymphocyte subgroup in the peripheral blood of mice were examined using flow cytometry. Compared with the model group, the four monomers could inhibit the increase in the proportion of CD8^+^ cells (*p* < 0.001 or *p* < 0.01) and the decrease in the proportion of CD4^+^ cells (*p* < 0.001 or *p* < 0.01), with a tendency to inhibit the decrease in CD4^+^/CD8^+^ (*p* > 0.05). CV, PC, and EC inhibited the reduction of the CD3^+^ cells ratio (*p* < 0.001) (Figure 5g–j). In influenza virus infection, the T cells level is an important indicator of the body's immune function. CD3^+^ is a common marker for all mature T cells, whether CD4^+^ helper T cells or CD8^+^ cytotoxic T cells, expressing CD3^+^. It plays an indispensable role in T cell antigen recognition, signal transduction, maturation, and immune regulation, and is a core component of T cell immune function (Mariuzza et al. 2020). The change in CD4^+^/CD8^+^ ratio can serve as a reference indicator for evaluating the severity and prognosis of influenza virus infection. A lower CD4^+^/CD8^+^ ratio may indicate more severe immune system damage and require close monitoring and timely treatment (Dengler et al. 2014). The experimental results showed a decrease in the proportion of CD3^+^ cells and CD4^+^/CD8^+^ ratio, indicating that influenza virus infection led to impaired immune function in mice, and the four monomers had a certain regulatory effect on the immune function of infected mice.

### 3.6 Antiviral Effects of PEC and PECP Formulation Groups In Vitro

**FIGURE 6:**
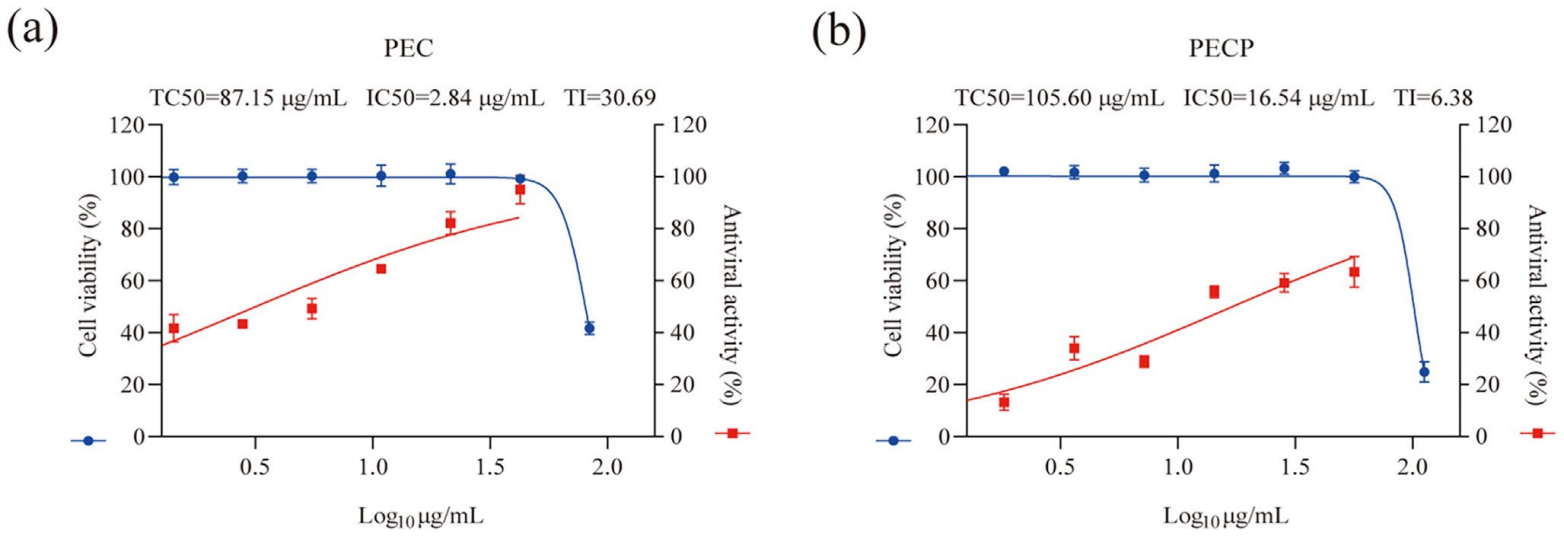
In vitro assays. Cytotoxicity and antiviral activity of (a) the formulation of PA, EC, and CV (PEC), (b) the formulation of PA, EC, CV, and PC (PECP) in MDCK cells (*n* = 3).

In vitro, the TI of the formulation decreased after the addition of PC, and its addition may lead to a decrease in both the effectiveness and safety of the formulation. Therefore, two formulations of PEC and PECP (PEC: PA 44.47 μg/mL, CV 30.04 μg/mL, EC 15.42 μg/mL; PECP: PA 44.47 μg/mL, CV 30.04 μg/mL, PC 26.84 μg/mL, EC 15.42 μg/mL) were designed for comparison to verify whether the addition of PC affected the overall efficacy of the formulations. The results showed that the cytotoxicity of both PEC and PECP was significantly reduced after the formulation compared to monomers, and the inhibitory effect of PEC on the virus was significantly higher than that of monomers. The therapeutic indices of PECP and PEC formulations against influenza virus were 6.38 and 30.69, respectively (Figure 6). The study found that PEC and PECP formulations showed promising antiviral effects in in vitro cellular assays. Based on this finding, we would explore their protective effects in a mouse model of influenza pneumonia.

### 3.7 Effects of PEC and PECP on the Survival of H1N1‐Infected Mice

**FIGURE 7:**
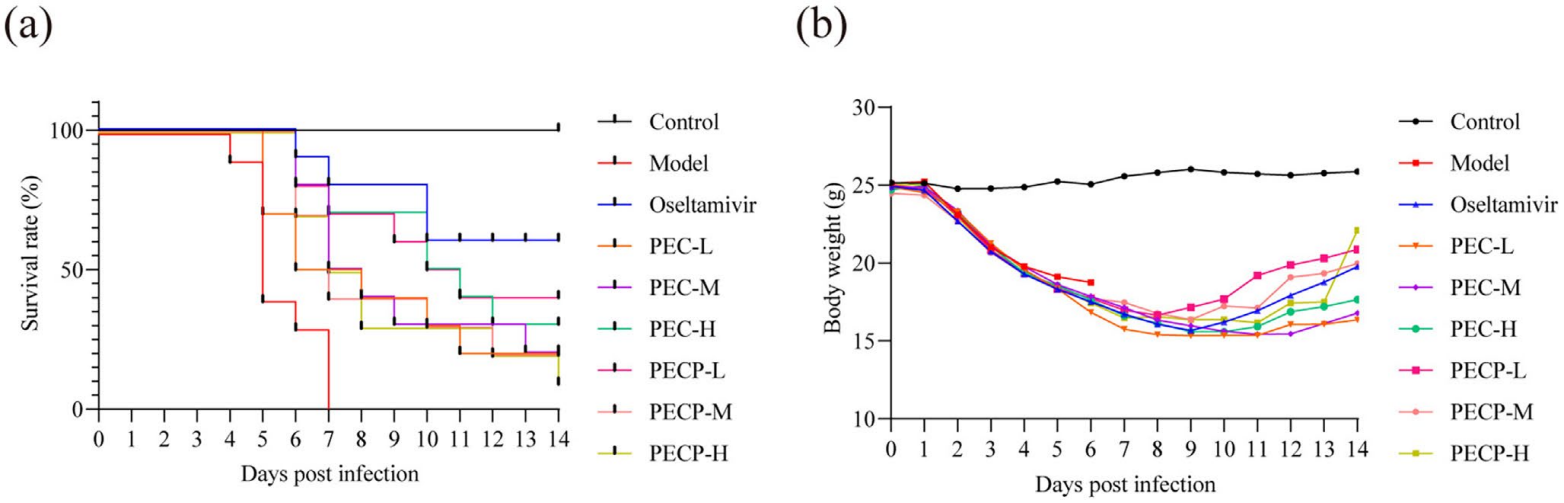
Survival test results of formulations. (a) Effect of different groups on the survival rate of H1N1‐infected mice (*n* = 10). (b) Average body weight changes of mice among different groups during the survival test.

This part of the experiment explored the death‐protective effects of three dose groups of PEC and PECP in H1N1‐infected mice. The same group of mice was used for this part of the experiment as for the monomers, so the data for the control, model, and oseltamivir groups overlapped. The results showed that the survival rates of mice in the PEC‐L, PEC‐M, PEC‐H, PECP‐L, PECP‐M, and PECP‐H groups were 20%, 20%, 30%, 40%, 20%, and 10%, respectively, after 14 days of infection with a lethal dose of H1N1 influenza virus (Figure 7). This demonstrated that both PEC and PECP groups were able to prolong the survival time and enhance the survival rate of mice to different degrees.

### 3.8 Protective Effects of PEC and PECP Formulations Against Acute Lung Injury Caused by H1N1 Infection

**FIGURE 8:**
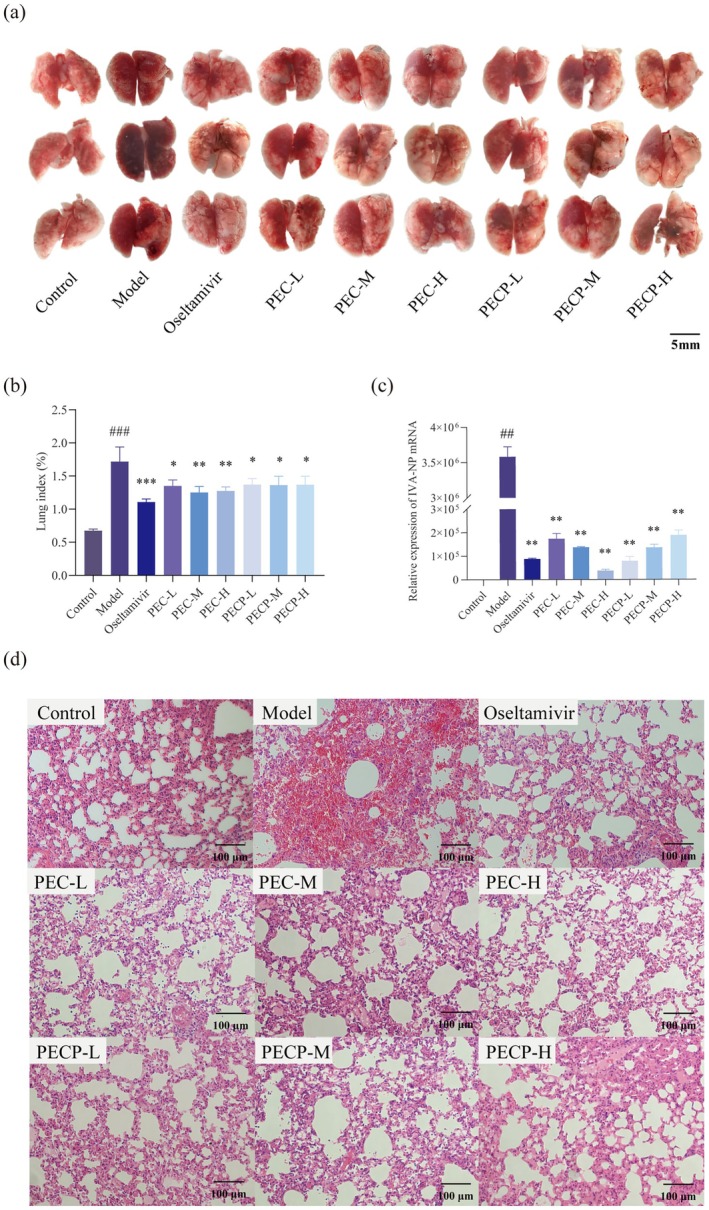
Effects of PEC and PECP on lung in H1N1‐infected mice. (a) Gross macroscopic examination of lungs in different groups. Changes of (b) lung index (*n* = 10) and (c) relative expression of IVA‐NP mRNA (*n* = 3) in lung among groups. (d) Pathological changes of lung tissues in mice following H&E staining. Data are presented as the means ± SD. *p < 0.05, **p < 0.01, ***p < 0.001 versus the model group. #*p* < 0.05, ##*p* < 0.01, ###*p* < 0.001 versus the control group.

Some lung indices can help to investigate the protective effects of the two formulations on the lungs of infected mice in vivo. The pulmonary viral load in this experiment is the relative expression of the influenza virus NP gene, which can indirectly reflect the amount of influenza virus in the lung tissue of mice. The experimental results showed that among all dosing groups, PEC‐H and PECP‐L had the most significant effect in reducing pulmonary viral load, but PECP‐L had a greater dose advantage over PEC‐H. Lung index and lung lesion results showed no significant differences among all dosing groups (Figure 8). As can be seen in the pictures of lung pathological changes, the lung tissue in the PEC and PECP groups macroscopically showed a lighter color, which implied fewer hemorrhagic points. The reduction of alveolar and endobronchial ventilation space was suppressed after administration of the drug, and all administration groups were able to alleviate pulmonary hemorrhage, inflammatory exudation, and lung consolidation to some extent. PECP was indeed less effective in vitro than PEC, but the experiment in vivo showed that the addition of PC to the formulation did not significantly reduce the efficacy, probably due to the more prominent effect of PC in anti‐inflammatory and immune function modulation. Considering the overall effect of the formulation on the postinfected organism, it was decided that PECP would be used as a research object in the formulation for subsequent studies.

### 3.9 Study of PECP Formulation Group on HINI‐Infected Mice Based on Regulation of Immune Function

**FIGURE 9:**
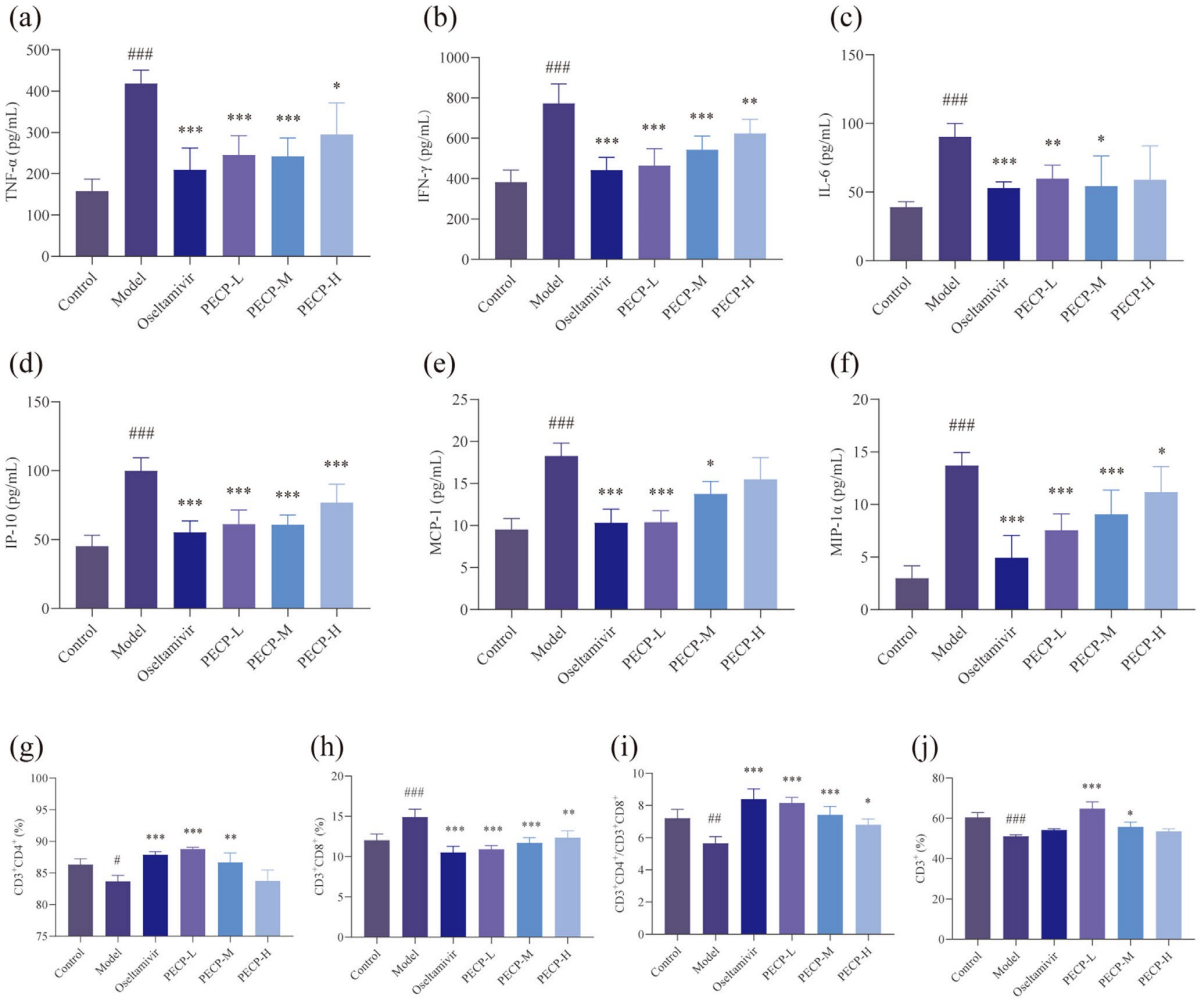
Effects of PECP on levels of cytokine/chemokine and the expression of the T lymphocyte immunophenotype in blood in H1N1‐infected mice. Levels of (a) TNF‐α, (b) IFN‐γ, (c) IL‐6, (d) IP‐10, (e) MCP‐1, (f) MIP‐1α in serum were determined using ELISA kits (*n* = 7). Changs in (g) CD3^+^CD4^+^/CD3^+^CD8^+^ and the percentage of (h) CD3^+^CD4^+^, (i) CD3^+^CD8^+^ and (j) CD3^+^ in blood between groups were detected by flow cytometry assay (*n* = 3). Data are presented as the means ± SD. *p < 0.05, **p < 0.01, ***p < 0.001 versus the model group. #*p* < 0.05, ##*p* < 0.01, ###*p* < 0.001 versus the control group.

The anti‐inflammatory and immunomodulatory effects of PECP on postinfected mice were shown in Figure 9. The results of ELISA showed that PECP was able to significantly inhibit the elevation of cytokines TNF‐α, IFN‐γ, and IL‐6 and chemokines IP‐10, MCP‐1, and MIP‐1α after infection in mice. The results of flow cytometry showed that PECP significantly inhibited the reduction of CD3^+^ and CD4^+^ cell ratios, the reduction of CD4^+^/CD8^+^, and the elevation of CD8^+^ cell ratios in mice after infection. During the hyperacute phase of viral infection, the cytokine storm has a significant impact on the destruction of CD4^+^ T cells and the immune system. This immune activation process may be the cause of immune system dysfunction in some individuals (Huang et al. 2023). In the early and middle stages of influenza virus infection, the cytokine storm caused autoimmune damage, leading to the destruction of immune function and overactivation, while PECP could produce an antagonistic effect and regulate mouse immune function. Among the three dose groups, the effect of PECP‐L was the most prominent (*p* < 0.001). It can be assumed that PECP inherited the advantages of PC in anti‐inflammation and immunomodulation.

### 3.10 Study on the Role of Volatile Monomers and Formulation Groups on the Mechanism of Inflammation Regulation in H1N1‐Infected Mice

**FIGURE 10:**
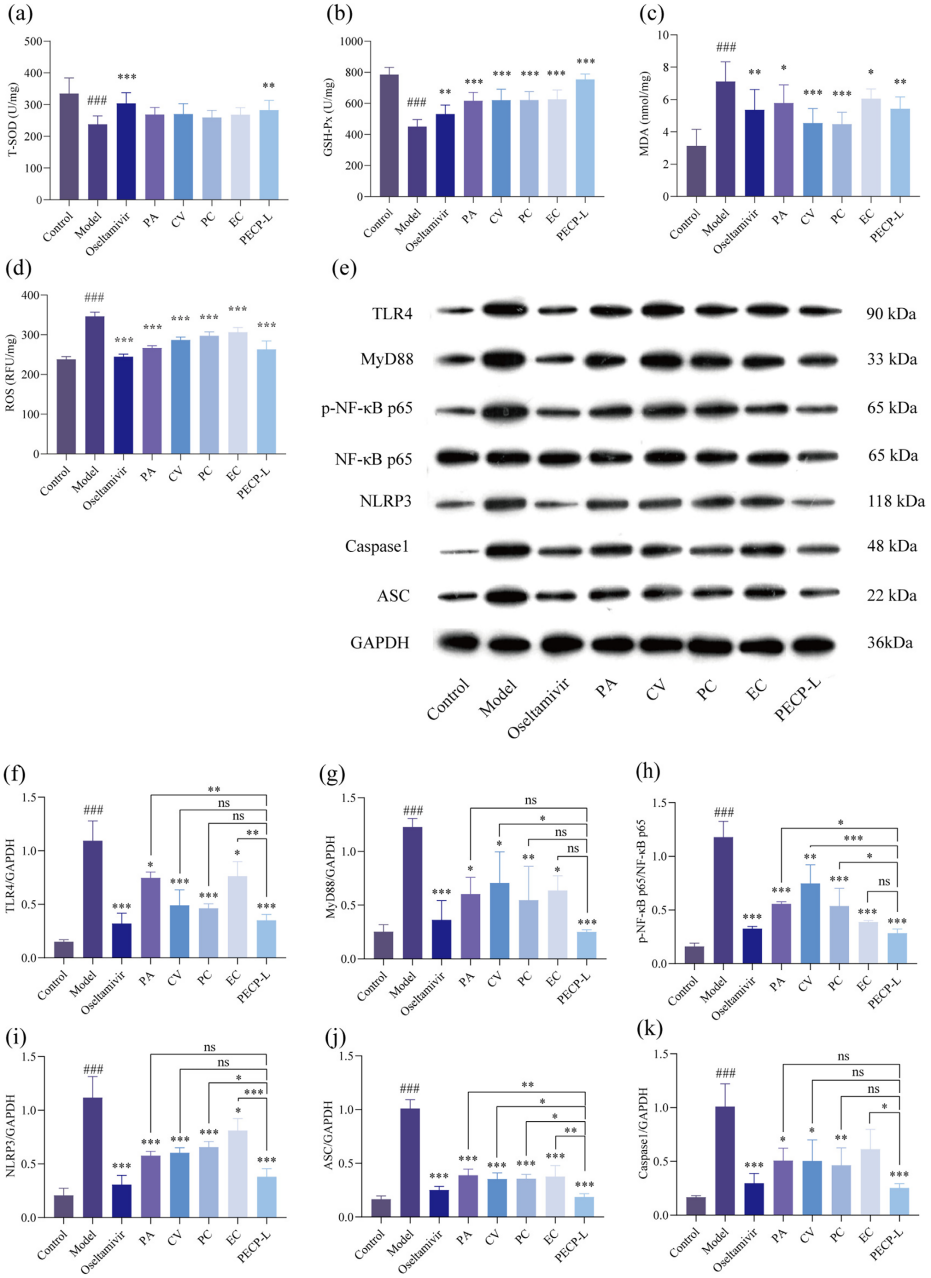
Effects of PA, CV, PC, EC, and PECP on antioxygenic ability and protein expression of TLR4/NF‐κB/NLRP3 signaling pathway in lung tissue in H1N1‐infected mice. Levels of (a) total superoxide dismutase (T‐SOD), (b) glutathione peroxidase (GSH‐Px), (c) Malondialdehyde (MDA), (d) reactive oxygen species (ROS) in lung tissue were determined using ELISA kits (*n* = 7). (e) Protein expression of TLR4, MyD88, p‐NF‐κB p65, NF‐κB p65, NLRP3, Caspase1, ASC in lung tissue determined by western blotting (*n* = 3). Quantification of (f) TLR4, (g) MyD88, (i) NLRP3, (j) Caspase1, (k) ASC protein expression relative to GAPDH. Quantification of (h) p‐NF‐κB p65 protein expression relative to NF‐κB p65 (*n* = 3). Data are presented as the means ± SD. *p < 0.05, **p < 0.01, ***p < 0.001 versus the model group. #*p* < 0.05, ##*p* < 0.01, ###*p* < 0.001 versus the control group.

The above research found that four volatile monomers and their formulations had anti‐inflammatory effects. There is a mutually reinforcing relationship between oxidative stress and inflammatory response. The following experiments explored whether four volatile monomers and their formulations can block the overexpression of ROS and inhibit the further occurrence of inflammation. T‐SOD and GSH‐Px are important antioxidant enzymes in the body that protect cells from oxidative damage. MDA is one of the end products of cell membrane lipid peroxidation, which reflects the degree of oxidative damage to cells. ROS can trigger oxidative stress and inflammatory reactions, and excessive ROS can cause direct damage to cells. As shown in Figure 10a–d, both the monomer and formulation could inhibit the production of ROS and MDA and increase the GSH‐Px level in lung tissues. In addition, the PECP‐L formulation could increase the T‐SOD level and protect the organism from oxidative damage.

To further validate the expression of proteins associated with anti‐inflammation, the TLR4/NF‐κB/NLRP3 pathway was selected to explore whether the drugs had any effect on the expression of key functional proteins in this pathway. Figure 10e–k showed the expressions of TLR4, MyD88, p‐NF‐κB p65, NF‐κB p65, NLRP3, Caspase1, ASC, and their relative gray values compared with the control protein (GAPDH) in different groups. Their relative gray values were up‐regulated in infected mice compared to the control group (*p* < 0.05), but reduced in both the monomer and formulation group (*p* < 0.05), with a more pronounced reduction in the formulation group. Therefore, these key functional proteins were validated to exert anti‐inflammatory effects on infected mice by regulating the TLR4/NF‐κB/NLRP3 pathway with monomers and formulations.

## 4 Discussion

The study focuses on the investigation of the pharmacodynamic effects and mechanisms of monomers of traditional Chinese medicines and their formulations on influenza virus pneumonia, with its experimental results mainly elaborated in four aspects like antiviral effects, regulation of immune function, antioxidant damage, and anti‐inflammation. Firstly, the TI of the drugs detected by in vitro cellular experiments reflected the toxicity‐efficacy relationship and antiviral effects of the drugs (Muller and Milton 2012). The animal survival experiment with a lethal dose of H1N1 infection revealed that all four monomers increased the survival rate of mice to different degrees. The previous study has shown that the survival rate, changes in body weight, and the status of mice after lethal infection by the influenza virus are the most intuitive indicators to assess antiviral efficacy (Song et al. 2016). Survival results showed that PA, CV, and EC probably could increase the survival rate, prolong the lifespan of H1N1‐infected mice, and inhibit their body weight loss. NP is a nuclear protein of the influenza virus that mainly regulates transcription and replication of the viral genome, which could indicate the number of influenza viruses in the host (Ma et al. 2018). The results showed that both monomers and formulations can reduce the level of NP, thus inhibiting the replication and proliferation of H1N1. In addition, both monomers and formulations could improve lung pathology in H1N1‐infected mice, inhibit the production of inflammatory cytokines and chemokines, and alter the ratio of T‐lymphocyte subgroups. T‐lymphocyte subgroups reflect the immune status of the organism, and an increase in CD4^+^/CD8^+^ suggests an improvement of drugs on the excessive immune response (Tsang et al. 2022; Graham et al. 1991), which all suggest that monomers and formulations have anti‐inflammatory and immunomodulatory effects.

Volatile components of aromatic Chinese herbs can be administered nasally, orally, or transdermally. Clinically, most Chinese medicine prescriptions and patent drugs are oral. A study showed oral use of patent Chinese medicine containing aromatic herbs' volatile oils was most common (Wang et al. 2014). Most of these components are irritating and highly volatile. The use of new processes and formulations may improve oral stability and bioavailability (Luo et al. 2021). Therefore, the oral mode of administration was chosen in this study. On the one hand, it is consistent with the traditional way of taking aromatic Chinese medicines in clinic. On the other hand, it can determine the efficacy of the drug first. This provides a research basis for the optimization of the dosage form in the subsequent development of new drugs. In the experiment on the protective effect of oral PA against influenza virus infection, the administration dose was 20 mg/kg–80 mg/kg, in which the middle‐dose group (40 mg/kg) and the high‐dose group (80 mg/kg) were more effective in inhibiting the mRNA expression of viral M genes, the regulation of different cytokines, and the promotion of immunoglobulin production (Li et al. 2012). CV can inhibit the excessive immune response induced by influenza A virus by suppressing virus replication and TLR/LRR pattern recognition, with an oral dose of 50 mg/kg (Zheng et al. 2021). EC extract is registered and licensed in Germany for the treatment of acute and chronic bronchitis, sinusitis, and respiratory tract infections at a dosage of 600 mg/d for adults (Juergens et al. 2003). Its extract has a protective effect on influenza virus pneumonia in mice, with oral doses of 30, 60, and 120 mg/kg. The effect was most significant at a high dose of 120 mg/kg (Li et al. 2016). There are no animal experiments on PC against influenza viruses. However, a drug for treating chronic bronchitis using PC as the raw material (*p*‐Cymene Soft Capsules) has been launched in China, with a daily dosage of 80 mg to 120 mg for adults. Therefore, based on the oral administration dosage range of the four monomers mentioned above, this experiment further determined the doses through the survival test.

The cytokines selected for detection in this study have a certain correlation with acute lung injury caused by influenza virus pneumonia. TNF‐α can inhibit the synthesis of early viral proteins to suppress virus replication, induce the generation of inflammatory mediators, and promote the aggregation of neutrophils in the lungs by increasing vascular permeability (You et al. 2021; Savenkova et al. 2024). IFN‐γ has antiviral effects and can promote the activation of various immune cells, enhance antigen presentation, and increase the activity of macrophage lysosomes (Seldeslachts et al. 2024; Malik et al. 2023). It can also induce macrophages to produce inducible nitric oxide synthase (iNOS), promote NO synthesis, and aggravate lung injury (Fu et al. 2023). IL‐6 rises sharply in the early stages of infection, promotes T cell activation and proliferation, B cell differentiation, and induces the production of autoantibodies (Hirano 2021; Aliyu et al. 2022). IP‐10, MCP‐1, and MIP‐1α are chemokines that aggregate white blood cells swimming out of blood vessels to the inflammatory focus, and also induce the secretion of pro‐inflammatory cytokines, such as IL‐1β, TNF‐α, and IL‐6, which accelerate the progression of local inflammatory responses (Mulla et al. 2022).

Interestingly, the study found that there were differences in the efficacy in vivo and in vitro. The validation in vivo is generally considered to be closer to the clinical efficacy (Bouhaddou et al. 2020). There are some possible reasons for this. Firstly, the experiments in vitro are directly targeting cells. The route of administration in vivo is oral, after absorption, distribution, and metabolism resulting in a low concentration of the drug at the site of the lesion, leading to poor bioavailability. Moreover, many candidate drugs with antiviral activity in vitro experiments only have a small fraction that can work in animals or humans, possibly due to systemic mechanisms in the body that can compensate for blocked target effects. For example, when a drug blocks an enzyme that the virus relies on for replication, the body may compensate for this blocked link by activating other related enzymes or metabolic pathways, allowing the virus to still replicate to a certain extent, thereby weakening the antiviral effect of the drug. This compensatory mechanism is a self‐protection response generated by the body to maintain its normal physiological functions and stable internal environment. However, in the case of drug treatment, it may lead to the inability of drugs that were originally effective in vitro experiments to achieve the expected antiviral effect in vivo (Zheng et al. 2022). These problems above need to be solved by some means in the future.

With regard to the experiments in vivo, due to the different dosages administered for the monomers and the formulations, it is not possible to compare the differences in the pharmacodynamic effects between these two in this study for the time being. However, the results of the pharmacodynamic experiments of the formulations proved that the combinations of several monomers can provide better therapeutic effects in terms of alleviating lung injury, inflammatory response, oxidative stress, and modulating immunity. There are synergistic effects among the components inside the formulations in most cases. Since the four monomers have different directions of therapeutic effects highlighted in the influenza pneumonia model of the monomer, such as the significant anti‐inflammatory effect of PC and the significant anti‐viral effect of PA and EC, etc. The formulated groups were proved to have significant advantages in antiviral and anti‐inflammatory, as well as immunomodulation, so the synergistic effect among the several monomers existed. Unfortunately, this study can only prove this conclusion at the macro level preliminarily, and more experiments are needed to supplement it. From the results of the pharmacodynamic experiments, it could be seen that there was a dose dependence in the PEC groups, but no dose dependence in the PECP groups, which is probably due to some problems with drug solubility in the high‐dose group of PECP. The monomers are all weakly water‐soluble oily substances, so the proportion of cosolvents for oral administration is limited, resulting in the separation of the drug from the water phase and failing to play the proper therapeutic effect in a better way. In the subsequent study, the dosage form of PECP should be optimized to achieve a better therapeutic effect.

In antiviral pneumonia research, the TLR4/NF‐κB/NLRP3 pathway is crucial. TLR4 can recognize the influenza virus and activate NF‐κB, which can upregulate pro‐inflammatory cytokines such as TNF‐α and IL‐6 (Shi et al. 2023; Yu et al. 2020). The activation of NF‐κB, combined with influenza‐related damage—associated molecular pattern (DAMP) and viral components as signals, can fully activate the NLRP3 inflammasome, which can release more inflammatory factors and worsen inflammation (Barnett et al. 2023; Ramachandran et al. 2024). Regulating this pathway can alleviate inflammation and reduce lung injury (Li et al. 2025; Chen et al. 2024).

Oxidative stress and inflammatory responses have mutually reinforcing effects, which are associated with the regulation of the TLR4/NF‐κB/NLRP3 pathway. Inflammatory factors activated through the immune response can produce a large number of ROS. At the same time, the cellular energy demand will gradually increase with the activation and differentiation of immune cells, and the production of free radicals will also increase synchronously with the enhancement of metabolism (Hu et al. 2022). Oxidative stress after breaking the redox balance can activate the inflammatory factor pathway, further producing a large number of inflammatory factors, which can ensure the continuation of the immune response and eliminate the invasion of pathogens. However, the over‐excited immune response turns the defense mechanism into an injury pathway to aggravate body damage (Lugrin et al. 2014). During microbial infections, the NLRP3 inflammasome helps the host immune system fight viruses, with its activation closely related to ROS (Alfonso‐Loeches et al. 2014). In addition, it has been shown that the TLR4/NF‐κB signaling pathway is also associated with oxidative stress (Dai et al. 2017).

Therefore, the study aims to elucidate the mechanism related to drug anti‐inflammation from the perspective of oxidative stress and the TLR4/NF‐κB/NLRP3 pathway. The results showed that monomers and formulations mainly achieved an improvement in influenza viral pneumonia through antiviral and anti‐inflammatory action. By inhibiting inflammatory pathways, reducing cytokine expression, and regulating inflammatory cells' infiltration, they improved the pulmonary inflammatory microenvironment and alleviated influenza pneumonia. However, there are still some limitations in the study. For example, the study just investigated the mechanism of action in terms of anti‐inflammation, which could not confirm the role of other targets and signaling pathways in the treatment of influenza with formulations composed of monomers. Therefore, further experimental validation from other aspects is needed in future studies.

## 5 Conclusion

The study investigated the efficacy of volatile monomer components and their formulations against influenza virus pneumonia caused by influenza A virus A/PR/8/34 (H1N1) infection, and comprehensively evaluated their efficacy from the inhibition of viral replication in vitro to the improvement of lung infection in vivo, the alleviation of “cytokine storm,” the anti‐oxidative stress, and immunomodulation, as well as investigated the efficacy of monomers and optimized the formulations and dosages. The study results showed that PA, CV, PC, and EC had certain inhibitory effects on influenza virus pneumonia, and their PECP formulation had the potential to be a therapeutic agent for influenza virus pneumonia, which could play a role in antivirus, antioxidative stress, regulation of immune function, and anti‐inflammation, with its mechanism may be related to the TLR4/NF‐κB/NLRP3 pathway.

## Conflicts of Interest

The authors declare no conflicts of interest.

## Supporting information


Appendix S1.

## Data Availability

The data that support the findings of this study are available on request from the corresponding author. The data are not publicly available due to privacy or ethical restrictions.

## Appendix A Screening of Volatile Monomer Components of Aromatic Herbs Against Viral Pneumonia

According to traditional Chinese medical science, viral pneumonias such as COVID‐19, SARS, and influenza viral pneumonia belong to the category of exogenous diseases in a broader sense, and are categorized as new‐emerging pulmonary epidemics of plague in a narrower sense (Wu and Wang 2012). New‐onset pulmonary epidemics are mainly caused by epidemics or epidemics combined with the six exogenous pathogenic factors, and have the ferocity and intractability of toxicity, as well as the specificity, strong contagiousness, and prevalence of epidemic qi (Zhao et al. 2023). In view of the irreplaceable role of traditional Chinese medicine (TCM), especially aromatic Chinese medicines, in the treatment of such diseases, antiviral pneumonia was used as an entry point in order to find antiviral pneumonia substances from aromatic Chinese medicines (ACM). Therefore, the search and screening of active ingredients for the treatment of viral pneumonia will be carried out through the pathway of “Clinical medication—Aromatic Chinese Medicines—Volatile oil components—Monomer components”

### Screening of ACM

#### Screening of ACM Against Viral Pneumonia in the Literature

##### Confirmation of the Importance of ACM in the Pattern of Medication in the Literature

We searched the literature on the use pattern of TCM for the prevention and treatment of viral pneumonia, counted the frequency of use of each medicine, and then calculated the proportion of ACM as a reflection of their importance.

1. Search scope: CNKI, CQVIP and Wanfang Data.
2. Search time: Until June 16, 2022
3. Search terms: CNKI with TKA = viral pneumonia AND TKA = traditional Chinese medicine AND TKA = regular patterns, Wanfang Data with Topic: viral pneumonia AND Topic: traditional Chinese medicine AND Topic: regular patterns, CQVIP with M = viral pneumonia AND M = traditional Chinese medicine AND M = regular patterns.
4. Search method: the literature was searched according to a predefined search strategy, using NoteExpress to eliminate duplicates and exclude literature not related to the study by reading the title and abstract.
5. Results: 149, 29, and 31 articles were retrieved, respectively; 156 articles remained after removing duplicates, and 90 articles remained after excluding noncompliant articles. Both retrieval and exclusion were conducted by two‐person mutual calibration.

The 90 papers were analyzed in depth, all of which were TCM interventions for the treatment or prevention of viral pneumonia and medication analysis. The frequency statistics of their selected traditional Chinese medicines indicate that a total of 258 kinds of traditional Chinese medicines were collected and a total of 3436 times used; the top 10 traditional Chinese medicines are shown in Table A1.

**TABLE A1:** Frequency of TCM in treating viral pneumonia in 90 papers after screening.

Serial number	Name of TCM	Frequency
1	Glycyrrhizae Radix Et Rhizoma	83
2	Pogostemonis Herba	83
3	Citri Reticulatae Pericarpium	81
4	Atractylodis Rhizoma	78
5	Poria	77
6	Scutellariae Radix	76
7	Atractylodis Macrocephalae Rhizoma	72
8	Armeniacae Semen Amarum	71
9	Forsythiae Fructus	70
10	Astragali Radix	68

As can be seen from Table A1, the percentage of Pogostemonis Herba, Citri Reticulatae Pericarpium, Atractylodis Rhizoma, Atractylodis Macrocephalae Rhizoma, and Forsythiae Fructus among the ACM was 384/759 = 50.59%, indicating that ACM occupy an important position in the treatment and prevention of viral pneumonia.

##### Secondary Search for ACM in the Literature

A secondary search for aromatic Chinese medicines against viral pneumonia.

1. Search scope: CNKI, CQVIP and Wanfang Data.
2. Search time: Until June 16, 2022.
3. Search terms: CNKI with TKA = viral pneumonia and TKA = aromatic, Wanfang Data with Topic: viral pneumonia and Topic: aromatic, CQVIP with M = viral pneumonia and M = aromatic.
4. Search method: literature was searched according to a predefined search strategy, and literature not related to the study was excluded by reading the title and abstract.
5. Results: 84, 13, and 111 articles were retrieved, respectively; 130 articles remained after removing duplicates, and 92 articles remained after excluding noncompliant articles. Both retrieval and exclusion were conducted by two‐person mutual calibration.

The 92 papers were analyzed in depth, and all of them were ACM for the treatment or prevention of viral pneumonia. Frequency statistics of the ACM appearing in the literature were conducted, and a total of 76 ACM were collected, which were used for a total of 1,288 times, and the top 40 aromatic herbs are shown in Table A2.

**TABLE A2:** Frequency of ACM in the treatment of viral pneumonia in 92 papers after screening.

Serial number	Name of ACM	Frequency
1	Pogostemonis Herba	85
2	Atractylodis Rhizoma	72
3	Citri Reticulatae Pericarpium	54
4	Magnoliae Officinalis Cortex	50
5	Acori Tatarinowii Rhizoma	48
6	Lonicerae Japonicae Flos	45
7	Forsythiae Fructus	44
8	Tsaoko Fructus	43
9	Atractylodis Macrocephalae Rhizoma	40
10	Angelicae Dahuricae Radix	38
11	Menthae Haplocalycis Herba	38
12	Bupleuri Radix	37
13	Ephedrae Herba	36
14	Asari Radix Et Rhizoma	36
15	Zingiberis Rhizoma Recens	34
16	Eupatorii Herba	33
17	Caryophylli Flos	33
18	Artemisiae Argyi Folium	30
19	Amomi Fructus Rotundus	28
20	Perillae Folium	27
21	Notopterygii Rhizoma Et Radix	26
22	Amomi Fructus	25
23	Borneolum Syntheticum	25
24	Cinnamomi Ramulus	24
25	Saposhnikoviae Radix	23
26	Cinnamomi Cortex	20
27	Moschus	20
28	Chuanxiong Rhizoma	19
29	Schizonepetae Herba	16
30	Dalbergiae Odoriferae Lignum	16
31	Euodiae Fructus	14
32	Aurantii Fructus Immaturus	13
33	Aucklandiae Radix	13
34	Asteris Radix Et Rhizoma	11
35	Artemisiae Annuae Herba	11
36	Santali Albi Lignum	11
37	Olibanum	11
38	Moslae Herba	10
39	Artemisiae Scopariae Herba	10
40	Houttuyniae Herba	9

#### Screening of ACM in Clinical Use Formulas for Antiepidemic

TCM has a long history of antiepidemic, in which ACM is widely used. Combining the widely used ancient antiepidemic prescriptions, the “Diagnosis and Treatment Protocol for Novel Coronavirus Pneumonia (Trial Version 9)” the “three TCM drugs and three herbal formulas” for antiepidemic, and the Chinese herbal prescriptions for the prevention and treatment of COVID‐19 by Profs. Wang Qi, Gu Xiaohong, and Wang Linyuan of Beijing University of Traditional Chinese Medicine, we will conduct a screening of ACM from the level of clinical application.

##### Screening in Ancient Formulas for Antiepidemic

1. Search scope: CNKI, CQVIP and Wanfang Data.
2. Search time: Until June 16, 2022.
3. Search terms: CNKI with (TKA = epidemic situation OR TKA = epidemic prevention) AND (TKA = ancient formulas OR TKA = famous formulas OR TKA = formulas recorded in classic medical writings before the Han Dynasty), Wanfang Data with (Topic: [epidemic situation] OR Topic: [epidemic prevention]) AND (Topic: [ancient formulas] OR Topic: [famous formulas] OR Topic: [formulas recorded in classic medical writings before the Han Dynasty]), CQVIP with (M = epidemic situation OR epidemic prevention) AND (M = ancient formulas OR famous formulas OR formulas recorded in classic medical writings before the Han Dynasty).
4. Search method: According to the preestablished search strategy, the literature was searched, and NoteExpress was utilized to weed out the weights and exclude the literature not related to the study by reading the title and abstract.
5. Results: A total of seven ancient formulas with the highest frequency of occurrence in antiepidemic were screened out, which were Huoxiang Zhengqi San, Dayuan Yin, Maxing Shigan Decoction, Maxing Yigan Decoction, Yin Qiao San, Yupingfeng San, Xuanbai Chengqi Decoction (Zeng et al. 2021; Su and Xiong 2021; Huang and Wang 2020).

##### Screening in Clinical Prescriptions of COVID‐19

With reference to the “Diagnosis and Treatment Protocol for Novel Coronavirus Pneumonia (Trial Version 9)”, the formulas for the diagnosis and treatment of COVID‐19 and Chinese patent medicines containing ATM were counted, including Lung Cleansing and Detoxification Decoction, Huashi Baidu Decoction, Cold‐Damp Plague Formula, Suhexiang Wan, and Angong Niuhuang Wan.

The clinical prescription of Beijing University of Traditional Chinese Medicine includes the prescription of Academician Wang Qi (Phragmitis Rhizoma, Lonicerae Japonicae Flos, Pogostemonis Herba, Rhodiolae Crenulatae Radix Et Rhizoma, Dryopteridis Crassirhizomatis Rhizoma, Polygoni Cuspidati Rhizoma Et Radix；Lonicerae Japonicae Flos, Phragmitis Rhizoma, Imperatae Rhizoma, Pogostemonis Herba, Angelicae Dahuricae Radix, Tsaoko Fructus), Professor Gu Xiaohong (Astragali Radix, Lonicerae Japonicae Flos, Forsythiae Fructus, Pogostemonis Herba, Atractylodis Rhizoma, Magnoliae Officinalis Cortex, Citri Reticulatae Pericarpium, Poria, Platycodonis Radix, Phragmitis Rhizoma；Pogostemonis Herba, Raphani Semen, Citri Reticulatae Pericarpium, Poria, Platycodonis Radix, Lonicerae Japonicae Flos, Forsythiae Fructus, Phragmitis Rhizoma, Scrophulariae Radix；Astragali Radix, Citri Reticulatae Pericarpium, Platycodonis Radix, Chrysanthemi Flos, Houttuyniae Herba, Pogostemonis Herba, Curcumae Longae Rhizoma, Poria, Mume Fructus, Glycyrrhizae Radix Et Rhizoma), Professor Wang Linyuan (Pogostemonis Herba, Moslae Herba, Polygonati Rhizoma, Perillae Folium, Tsaoko Fructus, Poria, Citri Reticulatae Pericarpium, Fici Hirtae Radix, Platycodonis Radix, Zingiberis Rhizoma Recens, Jujubae Fructus, Lonicerae Japonicae Flos, Eriobotryae Folium, Mori Folium, Raphani Semen).

By summarizing the above prescriptions, a total of 40 ACMs were screened out, as shown in Table A3.

**TABLE A3:** ACM in clinical usage formulas for antiepidemic.

Serial number	Name of ACM
1	Benzoinum
2	Atractylodis Macrocephalae Rhizoma
3	Angelicae Dahuricae Radix
4	Menthae Haplocalycis Herba
5	Borneolum Syntheticum
6	Atractylodis Rhizoma
7	Tsaoko Fructus
8	Bupleuri Radix
9	Aquilariae Lignum Resinatum
10	Citri Reticulatae Pericarpium
11	Chuanxiong Rhizoma
12	Caryophylli Flos
13	Saposhnikoviae Radix
14	Pogostemonis Herba
15	Cinnamomi Ramulus
16	Magnoliae Officinalis Cortex
17	Citri Grandis Exocarpium
18	Lonicerae Japonicae Flos
19	Chrysanthemi Flos
20	Forsythiae Fructus
21	Ephedrae Herba
22	Verbenae Herba
23	Aucklandiae Radix
24	Eupatorii Herba
25	Notopterygii Rhizoma Et Radix
26	Artemisiae Annuae Herba
27	Olibanum
28	Amomi Fructus
29	Moschus
30	Zingiberis Rhizoma Recens
31	Styrax
32	Santali Albi Lignum
33	Asari Radix Et Rhizoma
34	Cyperi Rhizoma
35	Moslae Herba
36	Cynanchi Paniculati Radix Et Rhizoma
37	Houttuyniae Herba
38	Aurantii Fructus Immaturus
39	Perillae Folium
40	Asteris Radix Et Rhizoma

### Screening of Volatile Oil Components of ACM

The main active ingredients of ACM are volatile components, and their volatile components mainly exist in the form of volatile oil. The search results of ACM in the literature were combined with the screening results of ACM in antiepidemic clinical use formulas to exclude the recurring categories of Chinese medicines, which were finally included in the screening scope of volatile oil components of Chinese medicines. The volatile oil fractions of ACM were traced, confirmed, and screened with a view to excavating the therapeutic material basis and searching for effective monomers.

The 40 ACMs in Table A1 were combined with the 40 ACMs in Table A3, and the total number was 30, which was used as the scope for the screening of volatile oils of ACM, as shown in Table A4.

**TABLE A4:** Screening scope of volatile oil components in ACM.

Serial number	Name of ACM
1	Atractylodis Macrocephalae Rhizoma
2	Angelicae Dahuricae Radix
3	Menthae Haplocalycis Herba
4	Borneolum Syntheticum
5	Atractylodis Rhizoma
6	Tsaoko Fructus
7	Bupleuri Radix
8	Citri Reticulatae Pericarpium
9	Chuanxiong Rhizoma
10	Caryophylli Flos
11	Saposhnikoviae Radix
12	Pogostemonis Herba
13	Cinnamomi Ramulus
14	Magnoliae Officinalis Cortex
15	Lonicerae Japonicae Flos
16	Forsythiae Fructus
17	Ephedrae Herba
18	Aucklandiae Radix
19	Eupatorii Herba
20	Notopterygii Rhizoma Et Radix
21	Artemisiae Annuae Herba
22	Olibanum
23	Amomi Fructus
24	Moschus
25	Zingiberis Rhizoma Recens
26	Asari Radix Et Rhizoma
27	Moslae Herba
28	Aurantii Fructus Immaturus
29	Perillae Folium
30	Asteris Radix Et Rhizoma

### Screening of Volatile Monomer Components of ACM

On the basis of the volatile oil components identified in “2 Screening of volatile oil components of ACM”, the top 3 monomer components by relative content of volatile oils of ACM were screened on the basis of the literature related to the compositional analysis of volatile oils, which are shown in Table A5.

**TABLE A5:** Top 3 monomer components by relative content of volatile oils of ACM.

Serial number	Name of ACM	Name of monomer component	References
1	Atractylodis Macrocephalae Rhizoma	Hinesol, Atractylolone, beta‐Guaiene	Pang et al. 2020
2	Angelicae Dahuricae Radix	alpha‐Pinene, Camphene, beta‐Phellandrene	Gao et al. 2016
3	Menthae Haplocalycis Herba	Menthol, Piperitone, Menthone	Aoken et al. 2021
4	Borneolum Syntheticum	Borneol	Chen et al. 2005
5	Atractylodis Rhizoma	beta‐Eudesmol, Hinesol, Atractylolone	Pang et al. 2020
6	Tsaoko Fructus	1,8‐Cineole, Geraniol, alpha‐Phellandrene	Quan 2016
7	Bupleuri Radix	n‐Heptane, Methylcyclohexane, (Z,Z)‐9,12‐Octadecadienoic Acid	Zhang et al. 2018
8	Citri Reticulatae Pericarpium	D‐Limonene, gamma‐Terpene, Myrcene	Zhu et al. 2021
9	Chuanxiong Rhizoma	Ligustilide, n‐Butylidenephthalide, beta‐Selinene	Liu et al. 2020
10	Caryophylli Flos	Eugenol, beta‐Caryophyllene, Eugenol Acetate	Jin et al. 2007
11	Saposhnikoviae Radix	Falcarinol, Octanal, D‐Limonene	Yang et al. 2015
12	Pogostemonis Herba	Patchouli alcohol, pogostone, alpha‐Bulnesene	Zhang et al. 2019
13	Cinnamomi Ramulus	trans‐Cinnamaldehyde, 4‐Methoxycinnamaldehyde, benzaldehyde	Gong et al. 2014
14	Magnoliae Officinalis Cortex	alpha‐Eudesmol, beta‐Eudesmol, gamma‐Eudesmol	He et al. 2012
15	Lonicerae Japonicae Flos	Linalool, 2‐Tridecanol, 1‐Tocene	Lin et al. 2020
16	Forsythiae Fructus	Palmitic Acid, 4 (14), 11‐Eucalyptus Diene, Nonadecanol	Wang et al. 2016
17	Ephedrae Herba	alpha‐Terpineol, Phytone, Phytol	Xue et al. 2020
18	Aucklandiae Radix	Dehydrocostus Lactone, Dihydroxylactone, Costunolide	Yang et al. 2019
19	Eupatorii Herba	Thymol, caryophyllene, carvacrol	Chen et al. 2017
20	Notopterygii Rhizoma Et Radix	alpha‐Pinene, beta‐Pinene, Terpinene	Tang et al. 2019
21	Artemisiae Annuae Herba	alpha‐Caryophyllene, caryophyllene oxide, eremophilene	She et al. 2011
22	Olibanum	Linalool, L‐Bornyl Acetate, ocimene	Zhang et al. 2016
23	Amomi Fructus	Bornyl Acetate, borneol, camphor	Ao et al. 2016
24	Moschus	Cineole, beta‐Bisabolene, D‐Limonene	Wang et al. 2008
25	Zingiberis Rhizoma Recens	Zingiberene, citral, beta‐Sesquiphellandrene	Li et al. 2013
26	Asari Radix Et Rhizoma	Safrole, Methyl Eugenol, Diethyl Phthalate	Wang et al. 2021
27	Moslae Herba	Thymol, carvacrol, *p*‐Cymene	Zheng et al. 2001
28	Aurantii Fructus Immaturus	D‐Limonene, gamma‐Terpinene, alpha‐Terpineol	Yuan et al. 2011
29	Perillae Folium	(‐)‐Perillaldehyde, D‐Limonene, caryophyllene	Li, Zheng, et al. 2018
30	Asteris Radix Et Rhizoma	Caryophyllene oxide, (‐)‐Humulene epoxide II, caryophyllene	Yuan et al. 2015

The number of relevant single components of the literature can reflect the research hotspots, so the number of relevant single components of the literature is the basis for rescreening.

1. Search scope: Web of Science, PubMed.
2. Search time: Until June 16, 2022.
3. Search terms: Web of Science used “TI = monomer name” and PubMed used “monomer name [Title]” as specialized search terms to search for relevant literature.
4. Search method: Direct search according to the predefined search strategy.
5. Results: Record the number of related literature and take the monomer components with the number of papers greater than 30, as shown in Table A6.

**TABLE A6:** Monomer components with a literature count greater than 30.

Serial number	Name of monomer component	Number of papers
1	Benzaldehyde	2424
2	Eugenol	1756
3	Menthol	1540
4	Palmitic acid	1335
5	n‐Heptane	1323
6	Thymol	1294
7	Carvacrol	1260
8	Camphor	1189
9	alpha‐Pinene	910
10	Citral	641
11	Linalool	598
12	Caryophyllene	465
13	Geraniol	451
14	*p‐*Cymene	443
15	Methylcyclohexane	349
16	Borneol	324
17	beta‐Pinene	314
18	Cineole	305
19	D‐limonene	257
20	1,8‐Cineole	253
21	Diethyl phthalate	242
22	Myrcene	184
23	trans‐Cinnamaldehyde	179
24	Ocimene	176
25	Camphene	173
26	Ligustilide	169
27	Phytol	164
28	alpha‐Terpineol	132
29	Menthone	108
30	Patchouli alcohol	83
31	Caryophyllene oxide	63
32	Octanal	40
33	alpha‐Phellandrene	38
34	beta‐Eudesmol	37
35	Pogostone	36

Screening of volatile monomer components needs to be focused on the antiviral dimension, especially against influenza virus and SARS‐CoV‐2, which are the most common pathogens causing viral pneumonia in recent years. Therefore, virus‐related content needs to be included in the search terms to further screen monomer components with antiviral activity.

1. Search scope: Web of Science, PubMed.
2. Search period: Until June 16, 2022.
3. Search terms: Web of Science with TI = monomer name AND TI = virus OR TI = monomer name AND TI = viral, PubMed with ((monomer name [Title]) AND (virus [Title])) OR ((monomer name [Title]) AND (virus [Title])) as the specialized search terms to search for relevant literature.
4. Search method: Direct search according to the predefined search strategy.
5. Results: The number of relevant monomer components of papers was recorded separately, and the papers related to influenza and COVID‐19 were selected out from them, and seven monomers were finally filtered out, as shown in Table A7.

**TABLE A7:** Number of papers related to anti‐virus monomers.

Serial number	Name of monomer component	Number of papers	Number of papers related to influenza and COVID‐19
1	Camphor	7	5
2	Carvacrol	6	2
3	Patchouli alcohol	6	6
4	Borneol	4	2
5	p‐Cymene	3	2
6	1,8‐Cineole	2	1
7	Camphene	1	1

As can be seen from Table A7, seven monomers including camphor, carvacrol, patchouli alcohol, borneol, p‐Cymene, 1,8‐Cineole, and Camphene, can be included in the selection. However, in the papers retrieved under the names of camphor, borneol, and camphene related to the resistance to influenza and COVID‐19, the substances with antiviral activity were their derivatives, not themselves, and were therefore excluded.

In summary, four monomers were screened that fit the direction of this study, namely patchouli alcohol, carvacrol, 1,8‐Cineole (eucalyptol), and p‐Cymene.

## Appendix B Steps for Drug Solution Preparation

### In Vitro

#### Preparation of Drug Solutions for the Four Volatile Monomers (PA, CV, PC, EC)

1. Preparation of masterbatch concentrations

The masterbatch concentration of 200 mM was prepared using DMSO before the experiment for immediate use.

2 Dilution of solutions

TC50 determination: The drug concentration was formulated to 200 μM using MEM medium diluted 1000‐fold and diluted at a 2‐fold ratio to obtain 8 concentration gradients.

IC50 determination: The drug concentration was formulated to 200 μM using virus culture solution (for the composition of the viral culture solution, see the contents of Main document 2.3) diluted 1000‐fold and diluted at a 2‐fold ratio to obtain 8 concentration gradients.

3 Unit conversion

To facilitate comparison of the in vitro antiviral activity of the monomers and their combinations, the units of concentration were all standardized to μg/mL, so that the maximum concentrations of the four monomers were 44.47 μg/mL (PA), 30.04 μg/mL (CV), 26.84 μg/mL (PC), 30.85 μg/mL (EC).

#### Preparation of Drug Solutions for PECP and PEC

1. PECP: A DMSO master mix containing PA (200 mM), CV (200 mM), PC (200 mM), and EC (100 mM) was prepared ready to use; the mixture was diluted 1000‐fold with MEM medium or virus culture solution at the time of use, and the total concentration of the mixture was 116.77 μg/mL (PA 44.47 μg/mL, CV 30.04 μg/mL, PC 26.84 μg/mL, EC 15.42 μg/mL), and 8 concentration gradients were done sequentially according to 2‐fold dilution.
2. PEC: A DMSO master mix containing PA (200 mM), CV (200 mM) and EC (100 mM) was prepared ready to use; the mixture was diluted 1000‐fold with MEM medium or virus culture solution at the time of use, and the total concentration of the mixture was 89.93 μg/mL (PA 44.47 μg/mL, CV 30.04 μg/mL, EC 15.42 μg/mL), and 8 concentration gradients were done sequentially according to 2‐fold dilution.

### In Vivo

The concentration of each medicinal solution is shown in Table A1. In order to prevent drug volatilization, each administration was prepared on the spot by dissolving four monomers and their combinations in DMSO, adding distilled water and Tween 80 to prepare a mixed solution (5% DMSO, 1% Tween 80). The solution remained stable and nonstratified during intragastric administration.
