# Supplementary material for: Inhibition Effects of Patchouli Alcohol, Carvacrol, p‐Cymene, Eucalyptol and Their Formulations Against Influenza Virus Pneumonia Through TLR4/NF‐κB/NLRP3 Signaling Pathway
Source: Chem Biol Drug Des. 2025 Aug 4;106(2):e70150. doi: 10.1111/cbdd.70150 (PMC12320473; doi:10.1111/cbdd.70150)
Supplement: Supplementary file 1 — Appendix S1. [file CBDD-106-e70150-s001.docx]

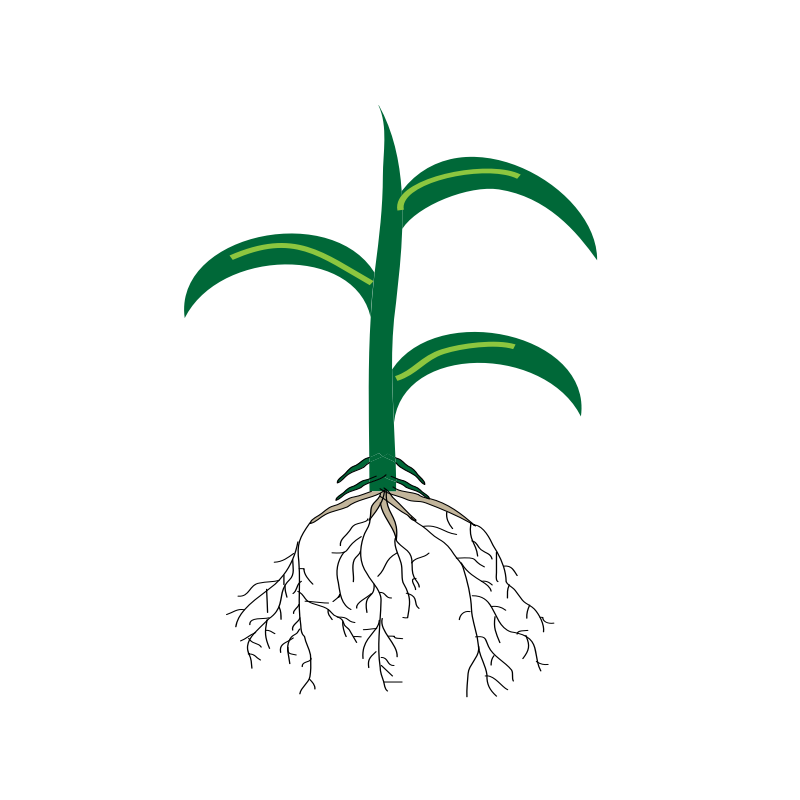


Maize_Plant icon by Guillaume Lobet https://figshare.com/authors/Plant_Illustrations/3773596 is licensed under CC-BY 4.0 Unported <https://creativecommons.org/licenses/by/4.0/>. This use does not make any changes to the illustration.


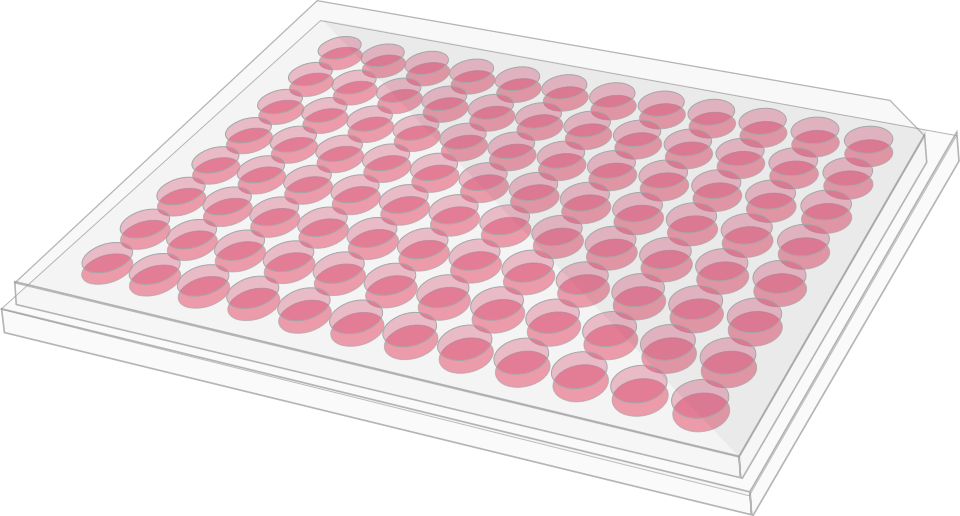


96_well_plate icon by Marcel Tisch https://twitter.com/MarcelTisch is licensed under CC0 <https://creativecommons.org/publicdomain/zero/1.0/>. This use does not make any changes to the illustration.


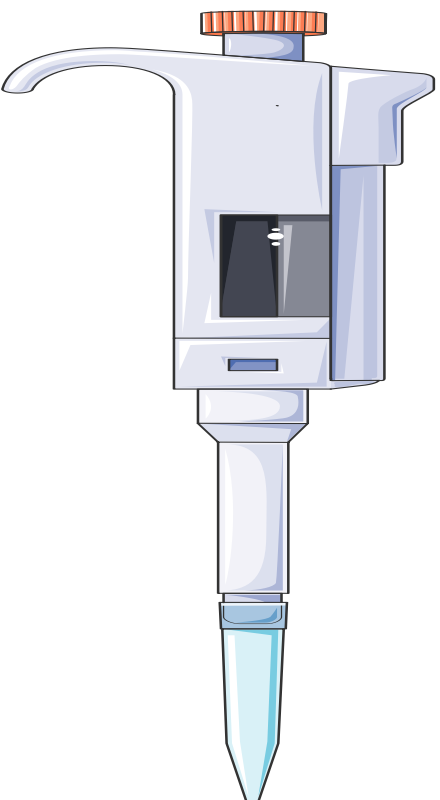


Micropipette icon by Servier https://smart.servier.com/ is licensed under CC-BY 3.0 Unported <https://creativecommons.org/licenses/by/3.0/>. This use does not make any changes to the illustration.


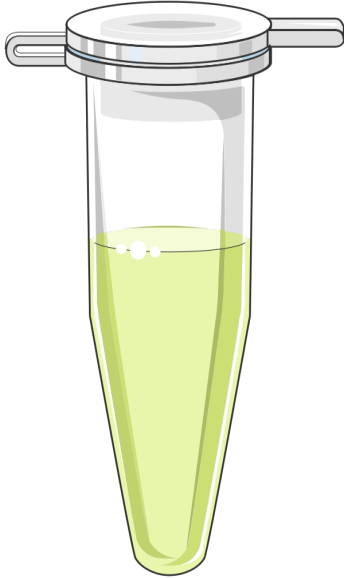


Microtube-closed-translucent icon by Servier https://smart.servier.com/ is licensed under CC-BY 3.0 Unported <https://creativecommons.org/licenses/by/3.0/>. This use does not make any changes to the illustration.


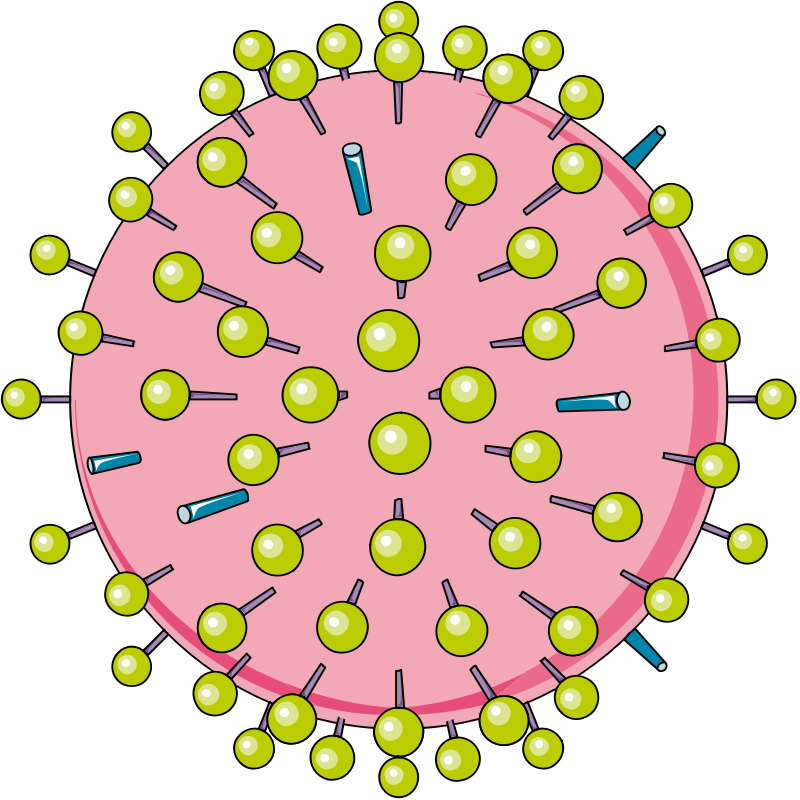


Influenza-virus icon by Servier https://smart.servier.com/ is licensed under CC-BY 3.0 Unported <https://creativecommons.org/licenses/by/3.0/>. This use does not make any changes to the illustration.


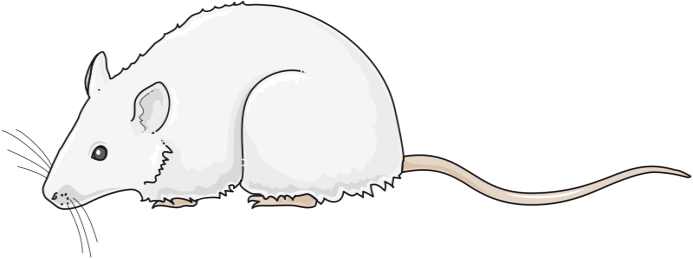


Rat-white icon by Servier https://smart.servier.com/ is licensed under CC-BY 3.0 Unported <https://creativecommons.org/licenses/by/3.0/>. This use does not make any changes to the illustration.


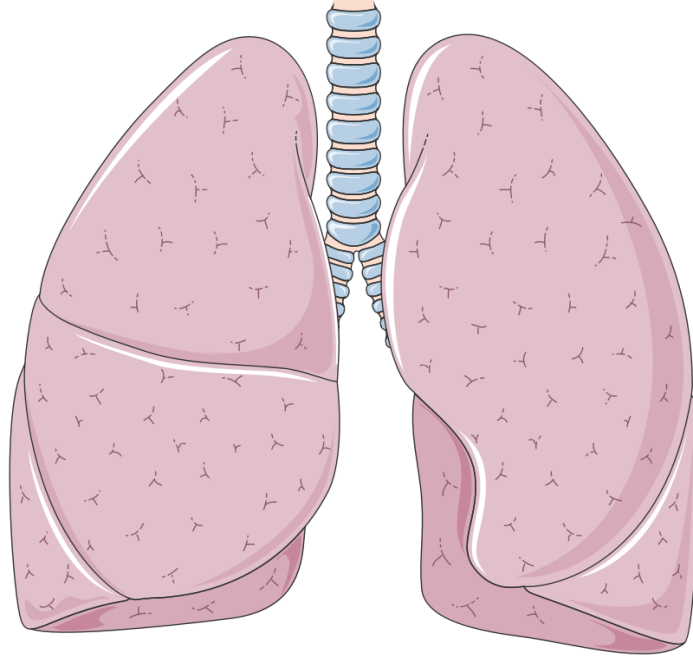


Lung icon by Servier https://smart.servier.com/ is licensed under CC-BY 3.0 Unported <https://creativecommons.org/licenses/by/3.0/>. This use does not make any changes to the illustration.


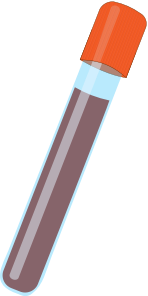


Blood_sample icon by Marcel Tisch https://twitter.com/MarcelTisch is licensed under CC0 <https://creativecommons.org/publicdomain/zero/1.0/>. This use does not make any changes to the illustration.
